# Supplementary material for: Maternal and child nutrition programme of investigation within the 100 Million Brazilian Cohort: study protocol
Source: BMJ Open. 2023 Sep 6;13(9):e073479. doi: 10.1136/bmjopen-2023-073479 (PMC10496662; doi:10.1136/bmjopen-2023-073479)
Supplement: Author's manuscript [file bmjopen-2023-073479.draft_revisions.pdf]

BMJ Open

BMJ Open is committed to open peer review. As part of this commitment we make the peer review history of every article we publish publicly available.

When an article is published we post the peer reviewers' comments and the authors' responses online. We also post the versions of the paper that were used during peer review. These are the versions that the peer review comments apply to.

The versions of the paper that follow are the versions that were submitted during the peer review process. They are not the versions of record or the final published versions. They should not be cited or distributed as the published version of this manuscript.

BMJ Open is an open access journal and the full, final, typeset and author-corrected version of record of the manuscript is available on our site with no access controls, subscription charges or pay-per-view fees (http://bmjopen.bmj.com).

If you have any questions on BMJ Open's open peer review process please email
info.bmjopen@bmj.com

BMJ Open

Maternal and child nutrition program of investigation within the 100 million Brazilian Cohort: study protocol

Journal:	*BMJ Open*
Manuscript ID	bmjopen-2023-073479
Article Type:	Protocol
Date Submitted by the Author:	06-Mar-2023

Complete List of Authors:	Carrilho, Thais; Federal University of Rio de Janeiro, Nutritional Epidemiology Observatory, Josué de Castro Institute of Nutrition<br>Silva, Natanael; Oswaldo Cruz Foundation, Centre for Data and Knowledge Integration for Health, Gonçalo Moniz Institute; University of Barcelona, Barcelona Institute for Global Health, Hospital Clínic<br>Paixão, Enny; London School of Hygiene & Tropical Medicine, Epidemiology and Population Health; Oswaldo Cruz Foundation, Centre for Data and Knowledge Integration for Health, Gonçalo Moniz Institute<br>Falcão, Ila; Oswaldo Cruz Foundation, Centre for Data and Knowledge Integration for Health, Gonçalo Moniz Institute; Federal University of Bahia, School of Nutrition<br>Fiaccone, Rosemeire; Federal University of Bahia, Institute of Mathematics and Statistics; Oswaldo Cruz Foundation, Centre for Data and Knowledge Integration for Health, Gonçalo Moniz Institute<br>Rodrigues, Laura; London School of Hygiene & Tropical Medicine, Department of Infectious Disease Epidemiology; Oswaldo Cruz Foundation, Centre for Data and Knowledge Integration for Health, Gonçalo Moniz Institute<br>Katikireddi, Srinivasa; University of Glasgow, MRC/CSO Social & Public Health Sciences Unit<br>Leyland, Alastair; University of Glasgow, MRC/CSO Social & Public Health Sciences Unit<br>Dundas, Ruth; University of Glasgow, MRC/CSO Social and Public Health Sciences Unit<br>Pearce, Anna; University of Glasgow, MRC/CSO Social & Public Health Sciences Unit<br>Velasquez-Melendez, Gustavo; Federal University of Minas Gerais, Department of Maternal and Child Nursing and Public Health, Nursing School<br>Kac, Gilberto; Federal University of Rio de Janeiro, Nutritional Epidemiology Observatory, Josué de Castro Nutrition Institute<br>Silva, Rita de Cássia; Oswaldo Cruz Foundation, Centre for Data and Knowledge Integration for Health, Gonçalo Moniz Institute; Federal University of Bahia, School of Nutrition<br>Barreto, Mauricio; Oswaldo Cruz Foundation, Centre for Data and Knowledge Integration for Health, Gonçalo Moniz Institute; Federal University of Bahia, Institute of Collective Health<br>Centre for Data and Knowledge Integration for Health, Nutrition Study Group (GeNut-CIDACS); Oswaldo Cruz Foundation, Centre for Data and Knowledge Integration for Health, Gonçalo Moniz Institute

Keywords:	NUTRITION & DIETETICS, Community child health < PAEDIATRICS, PUBLIC HEALTH

SCHOLARONE™
Manuscripts

Maternal and child nutrition program of investigation within the 100 million Brazilian Cohort: study protocol

Thais Rangel Bousquet Carrilho[1], Natanael de Jesus Silva[2,3], Enny S. Paixão[2,4], Ila Rocha Falcão[2,5], Rosemeire L. Fiaccone[2,6], Laura C. Rodrigues[2,4], Srinivasa Vittal Katikireddi[7], Alastair H. Leyland[7], Ruth Dundas[7], Anna Pearce[7], Gustavo Velasquez-Melendez[8], Gilberto Kac[1], Rita de Cássia Ribeiro-Silva[2,5*], Mauricio L. Barreto[2,9], on behalf of the Nutrition Study Group of the Center for Data and Knowledge Integration for Health (GeNut-CIDACS)

1. Nutritional Epidemiology Observatory, Josué de Castro Nutrition Institute, Federal University of Rio de Janeiro, Rio de Janeiro, RJ, Brazil.

2. Centre for Data and Knowledge Integration for Health, Gonçalo Moniz Institute, Oswaldo Cruz Foundation, Salvador, BA, Brazil.

3. Barcelona Institute for Global Health, Hospital Clínic, University of Barcelona, Barcelona, Spain.

4. Epidemiology and Population Health, London School of Hygiene and Tropical Medicine, London, UK.

5. School of Nutrition, Federal University of Bahia, Salvador, BA, Brazil.

6. Institute of Mathematics and Statistics, Federal University of Bahia, Salvador, BA, Brazil.

7. MRC/CSO Social & Public Health Sciences Unit, University of Glasgow School of Life Sciences, Glasgow, UK.

8. Department of Maternal and Child Nursing and Public Health, Nursing School, Federal University of Minas Gerais, Belo Horizonte, MG, Brazil.

For peer review only - http://bmjopen.bmj.com/site/about/guidelines.xhtml

9. Institute of Collective Health, Federal University of Bahia, Salvador, BA, Brazil.

Corresponding author:

Rita de Cássia Ribeiro-Silva. School of Nutrition, Federal University of Bahia. Av. Araújo Pinho, nº 32, Canela – Salvador, Bahia, Brazil / CEP: 40.110-150. Email: rcrsilva@ufba.br.

Collaborators of GeNut CIDACS:

The following are members of the GeNut-CIDACS – Nutrition Study Group of the Center for Data and Knowledge Integration for Health: Alastair H Leyland, Aline S Rocha, Anna Pearce, André A Mendes, Andrêa JF Ferreira, Audêncio Victor, Camila SS Teixeira, Carolina S Vieira, Craig Anderson, Dayana R Farias, Davide Rasella, Eduardo AF Nilson, Elizabete J Pinto, Enny S Paixão, Flávia JO Alves, Giesy R Souza, Gilberto Kac, Gustavo Velasquez-Melendez, Ila R Falcão, Jéssica Pedroso, Juliana F Mello e Silva, Laura C Rodrigues, Ligia Kerr, Marcia F Almeida, Maria YT Ichihara, Mariana S Felisbino-Mendes, Maurício L Barreto, Naiá Ortelan, Miriam H Tsunemi, Natanael J Silva, Nathalia S Guimarães, Rafaela S Andrade, Rita CO Carvalho-Sauer, Rita C Ribeiro-Silva, Rosemeire L Fiaccone, Ruth Dundas, Sara A Silva, Srinivasa V Katikireddi, Thais RB Carrilho, Walmir M Caminhas.

Word count - abstract: 298/300

Word count - main text: 3508

Number of references: 70

Number of figures: 1

Number of tables: 3

For peer review only - http://bmjopen.bmj.com/site/about/guidelines.xhtml

ABSTRACT

Introduction: There is a limited understanding of the early nutrition and pregnancy determinants of short- and long-term maternal and child health in ethnically diverse and socioeconomically vulnerable populations within low- and middle-income countries. This investigation program aims to: 1. describe maternal weight trajectories throughout the life course; 2. describe child weight, height, and body mass index (BMI) trajectories; 3. create and validate models to predict childhood obesity at five years of age; 4. estimate the effects of pre-pregnancy BMI, gestational weight gain (GWG), and maternal weight trajectories on adverse maternal and neonatal outcomes and child growth trajectories; 5. estimate the effects of pre-pregnancy BMI, GWG, maternal weight, and interpregnancy BMI changes on maternal and child outcomes in the subsequent pregnancy; and 6. estimate the effects of maternal food consumption and infant feeding practices on child nutritional status and growth trajectories. **Methods and analysis:** Linked data from four different Brazilian databases will be used: the 100 million Brazilian Cohort, the Live Births Information System, the Mortality Information System, and the Food and Nutrition Surveillance System. To analyze trajectories, latent-growth, superimposition by translation and rotation (SITAR), and broken stick models will be used. To create prediction models for childhood obesity, machine learning techniques will be applied. For the association between the selected exposure and outcomes variables, generalized linear models will be considered. Directed acyclic graphs will be constructed to identify potential confounders for each analysis investigating potential causal relationships. **Ethics and Dissemination:** This protocol was approved by Research Ethics Committee from the authors' institutions. The linkage will be carried out in a secure environment. After the linkage, the data will be de-identified, and pre-authorized researchers will access

For peer review only

the dataset via a virtual private network connection. Results will be reported in open-access journals and disseminated to policymakers and the broader public.

Keywords: Maternal nutrition, gestational weight gain, infant nutrition, growth charts, childhood obesity, nutritional status, food consumption, routinely collected health data.

ARTICLE SUMMARY

Strengths and limitations of this study

- The large sample size and diversity of populations to be used in this study, including data of minorities such as indigenous and *quilombolas* (descendants of people who were slaved), the possibility to stratify the analyses according to several intersecting socioeconomic characteristics, and the use of longitudinal data are unique aspects of this nutrition research program.
- The analytical approaches include cutting-edge methods to analyze growth trajectories, such as the SITAR and the broken stick models, and to create predictive models using machine learning techniques.
- Data from administrative datasets will be analyzed, which were not produced for research and, therefore, may be more prone to measurement errors when compared to data primarily collected for research purposes.
- The study is potentially vulnerable to linkage bias since data from several different administrative systems will be linked.
- The coverage of some national information systems is restricted to socioeconomically disadvantaged populations and varies in quality and completeness across regions of Brazil. This will enable estimates for specific groups, but results may not be generalizable.

For peer review only

INTRODUCTION

Maternal nutritional status before, during, and after pregnancy has been associated with the risk of adverse outcomes for the infant and the mother (1-4). Pre-pregnancy overweight, excessive gestational weight gain (GWG), and interpregnancy weight increase are associated with outcomes such as the birth of large for gestational age (LGA) infants, neonatal mortality, maternal gestational diabetes, hypertensive disorders, and mortality, and in the long-term, childhood and maternal obesity (1-13). Thus, the increase in the prevalence of such predictors among women of reproductive age in low- and middle-income countries (LMICs) may elevate the risk on the referred outcomes (14). In Brazil, more than 60% of adult women are overweight (15). Thus, the association between maternal nutritional status and adverse outcomes, especially in the medium and long-term, needs to be further explored.

The association of maternal interpregnancy weight change with outcomes in subsequent pregnancies can also be of great importance. However, few studies have explored this relationship due to the need for long-term follow-up (16). Studies show that interpregnancy increases in body mass index (BMI) may increase the risk of short-term outcomes, such as gestational diabetes, hypertensive disorders, cesarean delivery, and thromboembolism in the subsequent pregnancy (9, 17). It has also been shown that BMI reduction can increase the risk of small for gestational age (SGA) birth (17). However, little is known regarding the association of pre-pregnancy BMI, GWG, postpartum weight retention, BMI change during pregnancies, and important long-term adverse outcomes, such as maternal and child mortality and childhood obesity, in developing and socioeconomically vulnerable populations. In addition, maternal weight and child growth patterns and trajectories remain underexplored in these populations, mainly due to the

For peer review only - http://bmjopen.bmj.com/site/about/guidelines.xhtml

lack of longitudinal nationally representative data. It is crucial to identify those trajectories, their determinants, and how they are related.

Furthermore, studies investigating the influence of maternal and infant diet on the nutritional status of children under five based on national population data are also scarce and could help shape maternal and child health services and policy interventions. In general, most available studies evaluated the association between maternal and child diet. Bjerregaard et al. (18) studied 19,582 mother-offspring pairs in the Danish National Birth Cohort. They observed a significant association between the maternal diet quality during pregnancy and the quality of the offspring's diet at the age of 14. The results from another cohort, conducted among 3,422 mothers and children from Portugal, revealed that children whose mothers had worse dietary scores were more likely to follow unhealthier patterns at the age of four (19). Thus, maternal and infant diet seem to be related and to affect child's nutritional statis. However, further investigation to tease apart the complex pathways between the mother and infant's diet and child nutritional status is required.

The use of data from national administrative systems has increased throughout the years (20). The utilization of those data for research purposes has recently received attention with improving methods to analyze big data, enhanced secure storage infrastructure, and increased computational capacity (21, 22). Using data collected as part of the routine of healthcare services can be helpful to monitor trends in health indicators and inform public health policies (23). These data are critical in LMICs, where investment in research is generally scarce. Since 1975, several information systems have been created in Brazil. However, little has been done to harness their resources, especially in the maternal and child nutrition field. Big administrative databases can make it possible to study several maternal and child nutritional health problems which have only been investigated in studies with small sample sizes or not explored at all.

For peer review only

Maternal nutritional status, diet, and lifestyle, and the family's sociodemographic conditions are all potential determinants of the occurrence of maternal and infant adverse outcomes. However, these relationships were not adequately tested in LMICs. In addition, these are complicated pathways, and causal methods, applied to longitudinal data and through a life course lens, along with large sample sizes, can help in the understanding of those. Thus, the current protocol represents an unprecedented study in a LMIC population, and it differs from currently available studies carried out in small cohorts and with restricted geographical representation. To the best of our knowledge, this is the first initiative to use a population-based linkage database of administrative and health registry data to study maternal and child health and nutritional outcomes in Brazil.

Our objectives in this nutrition research program are to: 1. describe maternal body weight trajectories during the life course (adolescence, adulthood, pregnancy, and postpartum); 2. describe child weight, height, and BMI trajectories until the age of five; 3. create and validate models to predict childhood obesity at the age of five years; 4. Estimate the effects of pre-pregnancy BMI, GWG, maternal weight, and BMI changes between pregnancies, and maternal weight trajectories on adverse maternal and neonatal outcomes and child growth trajectories; 5. estimate the effects of pre-pregnancy BMI, GWG, and maternal weight and BMI changes on adverse maternal and neonatal outcomes in the subsequent pregnancy; 6. estimate the effects of maternal and infant feeding practices (food intake markers during pregnancy, breastfeeding, and complementary feeding) on child nutritional status and growth trajectories. We expect that the results obtained from these studies can be translated into actions to improve women's and children's health care and public health policy, and in the long term, in an improvement in the health indicators in these groups.

For peer review only

METHODS AND ANALYSIS

Conceptual framework

Our conceptual framework considers maternal nutritional status, diet, and lifestyle, and the family's sociodemographic conditions as potential determinants of the occurrence of adverse maternal and child outcomes (**Figure 1**). The complex relationships underlying this framework have been poorly investigated in LMICs. Most evidence regarding these associations comes from high-income countries or was conducted with small sample sizes in LMIC populations (3, 11, 24).

Data source and linkage process

This protocol was developed following the Reporting of Studies Conducted Using Observational Routinely Collected Health Data (RECORD) guidelines (25). This study will use linked data from four different Brazilian databases: the 100 Million Brazilian Cohort baseline, the Live Births Information System (SINASC, from the Portuguese acronym), the Mortality Information System (SIM), and the Food and Nutrition Surveillance System (SISVAN) (**Table 1**). The 100 Million Brazilian Cohort is a dynamic and open cohort created by the *Centro de Integração de Dados e Conhecimentos para Saúde* (CIDACS) (26). The cohort was designed to study the effect of social determinants and social policies on the health of Brazilian people, mainly those who are beneficiaries of social protection programs. It includes individual data from different national databases from January 2008 to December 2018. The cohort is based on the *Cadastro Único* (CadUnico), a shared registry for more than 20 Brazilian social programs, containing data from families with a monthly income ≤ of three minimum wages (~750 USD). The CadUnico database originates from an extensive questionnaire completed at the time of

For peer review only - http://bmjopen.bmj.com/site/about/guidelines.xhtml

application to federal social programs and contains detailed demographic, economic, and social data at the household and individual levels. The CadUnico database was used to generate the baseline of the 100 Million Brazilian Cohort. More details of this process can be found elsewhere (27, 28). The baseline registers of the cohort comprise 131,697,800 individuals.

The SINASC is the primary national system recording the data available in the "declaration of live birth", legally required for every live birth in Brazil. The declaration is filled in immediately after birth by the health worker who assisted the childbirth. In cases where the delivery was not assisted or performed with a registered professional, the declaration is filled in by the Civil Registry Office with the data provided by the declarant. The declaration form includes data on pregnancy and delivery. Details of the system can be found elsewhere (29). CIDACS has access to identified data of the SINASC between 2001 and 2018, comprising more than 52 million records.

The third database, the SIM, records data from the "declaration of death", which is a document that must be completed by a physician who attests to the cause of death. The form contains information on the identity of the deceased, their place of residency and death, and the cause of death. For infant mortality under 1 year of age, it also provides information on the deceased's mother. The details of the death certificates can be found elsewhere (30). The database available at the CIDACS includes 20,366,604 records between 2001 and 2018.

Finally, the fourth database is the SISVAN, which monitors the Brazilian population's nutritional status since 2008 (31). The system was designed to record individual sociodemographic, anthropometric, and food intake data of subjects using public healthcare services in all life-course stages. The main goal of the SISVAN is to inform the evaluation and development of public health nutrition policies. The dataset

For peer review only - http://bmjopen.bmj.com/site/about/guidelines.xhtml

available at CIDACS includes 307,245,508 records (59,724,164 individuals) in all the life cycles from 2008 – 2017. Women of all ages and children aged up to 10 years correspond to 76.4% (235,801,691) and 37.6% (115,510,621) of the records in the SISVAN.

For the work described in this protocol, we will link the 100 Million Cohort baseline data with the data from the SISVAN, SIM, and SINASC. The 100 Million Brazilian Cohort will form the population spine for linkage. Two approaches will be used to perform record linkage between the four sources: **a.** deterministic linkage; **b.** non-deterministic linkage based on the similarity index. Deterministic linkage will be performed between the 100 Million Brazilian Cohort baseline and the SISVAN because both datasets contain the Social Information Number (NIS), a unique identifier assigned for each individual. Linkage of the other administrative datasets will occur using a non-deterministic approach. This method will also link the data from the cohort with the subset of individuals in the SISVAN with no NIS. To do that, CIDACS created a tool (CIDACS-RL) to link individual records based on identifiers, such as name, sex, age or date of birth, mother's name, and the municipality of residence (32, 33). The CIDACS-RL applies indexing and searching algorithms implemented in the Apache Lucene solution as the blocking strategy. The indexation strategy allows the CIDACS-RL to search for the most similar records from the indexed dataset for each record in the main dataset and submit them to the pairwise comparisons step. Candidate linking records are ordered by the similarity scores, and only the comparison pair with the highest score is retained as a potential link. All remaining candidate records are discarded (32, 34).

The final linked dataset will be submitted to an extensive data structuring process before the analyses are performed. We will first identify the mother-child pairs in the 100 Million Brazilian Cohort baseline based on the child's family ID number and birth information, such as the mother's name and date of birth. This identification enables the

For peer review only - http://bmjopen.bmj.com/site/about/guidelines.xhtml

structuration of the dataset and the link between the mother and child data. This step is essential because the mother-child pairs are not automatically identified in the linkage.

Main variables

The main variables for analyses are summarized in **Table 2**. Several maternal and child variables can be created with the linked datasets. These variables will be analyzed as outcomes or exposures/confounders for several objectives proposed in this protocol. It is important to mention that, whenever possible, we plan to explore those variables as continuous and categorical, according to the proposed cutoffs. For example, children's BMI will be tested as a continuous score and categorized into underweight, normal, overweight, and obesity. Other essential variables to achieve the proposed objectives include socioeconomic and demographic conditions, newborn, mother, pregnancy, and delivery characteristics (**Table 1**).

Analytical approaches

The final linked dataset will be submitted to an extensive data-cleaning process before the analyses are performed. In this step, we plan to implement a routine to identify implausible anthropometric values considering cross-sectional and longitudinal data. The approaches proposed by Yang and Hutcheon (35), based on conditional centiles; Shi et al. (36), based on jackknife residuals; and the method based on linear mixed spline regression proposed by Welch et al. (37) and Boone-Heinone et al. (38) will be considered. In addition, modifications of the methods to deal with cross-sectional data (39) and methods to identify populational outliers based on *z* scores of reference charts will also be considered (38). After the identification, we plan to perform sensitivity

For peer review only

For peer review only - http://bmjopen.bmj.com/site/about/guidelines.xhtml

analyses to assess the impact of removing the observations flagged as outliers from the dataset.

Finally, because Brazil is a remarkably diverse country, we plan to perform a heterogeneity assessment of the main variables to be used in the studies across the states and regions to ensure we are working with homogeneous data. Two approaches will be considered in this step: a. the use of multilevel models to calculate the proportion of the variance in the variable of interest that could be attributable to the origin of the data (39), in this case, the region of residence; b. the standardized site difference method, as proposed by the WHO in the Multicentre Study (40). This evaluation of heterogeneity will also capture aspects related to the quality and coverage of the dataset, which will be considered before deciding the next steps of the analyses.

We plan to conduct descriptive analyses initially, including the presentation of summary measurements (mean, standard deviations, median, interquartile range, kurtosis, and skewness measurements) for continuous variables and absolute (n) and relative (%) frequencies for categorical ones. A summary of the proposed analytical approaches for each goal is available in **Table 3**.

Because we plan to work with large sample sizes, it will be possible to adopt an intersectionality perspective, exploring how combinations of social characteristics (such as gender and race/ethnicity or socioeconomic position and race/ethnicity) are related to outcomes of interest (41). We will do that by stratifying the analyses according to several variables, such as those identifying different social contexts. For example, we will be able to stratify the analyses by Brazilian municipality, identifying clusters of richer (closer to the European reality) and poorer (closer to Sub-Saharan Africa context) populations, and explore how combinations of family size, race/ethnicity, urban/rural context influence the outcomes.

For peer review only - http://bmjopen.bmj.com/site/about/guidelines.xhtml

To describe trajectories in maternal weight and child weight, height, and BMI, we plan to test three different classes of models: latent class growth, broken stick, and Super Imposition by Translation And Rotation (SITAR) models. The novelty of this study is the possibility of stratifying the trajectories according to several sociodemographic and economic variables, such as race/ethnicity and participation in conditional cash-transfer programs.

We plan to use latent-class growth models to identify trajectory groups that can be later associated with risk factors (42). Those models allow longitudinal data to inform clusters of individuals following different patterns of change over time, and they account for varying distances between the measurements. The number of trajectory groups can be selected based on Bayesian information criteria (42).

The *broken stick* models are another recent approach to be used to model growth trajectories. These models are a particular case of the linear mixed model. They summarize irregular individual trajectories, i.e., non-equidistant measurements over time, by estimates made at a pre-specified time interval (43). The models were initially developed to facilitate the statistical analysis and the testing of critical ages in the onset of childhood obesity, but several other applications are possible (43, 44).

Finally, we plan to test the SITAR models. These models describe each trajectory in three biologically interpretable parameters: size, time, and velocity. By using them, we may estimate each child's variation from the curve expressed by child-specific random effects estimates for absolute amount, timing, and acceleration (45). These three child-specific estimates can be used as exposure variables to explore associations with adverse outcomes (46). It is important to mention that those three techniques are used for different goals, which should be considered when choosing the final model. To select the final

For peer review only - http://bmjopen.bmj.com/site/about/guidelines.xhtml

model to create the trajectories that will be used in the subsequent studies, we will consider the group's existing knowledge and experts' opinions.

To construct predictive models for childhood obesity at the age of five, to identify factors that could be used to target interventions, we plan to consider artificial intelligence models, such as those based on Artificial Neural Networks and Adaptive Fuzzy Systems (47, 48). The risk and protective factors to be considered in these models are the characteristics of the child, characteristics of the mother, and family/environment factors. The following steps will be executed to create and validate the models: a. data aggregation and pre-processing (filtering and eliminating inconsistencies); b. data separation in sets of training, validation, and testing; c. training of the models; and d. results, validation, and testing of the models. The performance of the models will be evaluated through the measures of sensitivity, specificity, positive and negative predictive values, overall accuracy, and the area under the Receiver Operating Characteristic (ROC) curves. Experts will also validate the results based on documents and reference frameworks for child obesity assessment. We will also assess the performance of the algorithm for different population subgroups, particularly from an equity perspective, to identify how it works among more socioeconomically disadvantaged and minority ethnic groups.

To estimate the effects of pre-pregnancy BMI, GWG, maternal weight, and BMI changes between pregnancies, and maternal weight trajectories on the selected outcomes, we plan to use generalized linear models (GLMs). For categorical outcomes, logistic and Poisson regressions with robust variance will be considered. For continuous outcomes, linear regression will be used. We also plan to explore quantile regression for continuous outcomes. If the exposure is a longitudinal variable, we plan to account for the intra-subject variability by running linear mixed-effects models to extract the best linear unbiased prediction (BLUP). This longitudinal prediction of the exposures can then be

For peer review only - http://bmjopen.bmj.com/site/about/guidelines.xhtml

incorporated into a GLM model (49). Whenever the outcome is longitudinal, linear mixed-effects models will be used. We will consider the distribution of the variables and perform transformations whenever necessary. In some situations, we may also consider modeling the data with restricted cubic splines (50) or fractional polynomials (51, 52) to account for the non-linearity of the relationships between the given variables. After adjusting the models, we plan to run the proper diagnostics to evaluate the goodness of fit.

To define the set of confounders to be used in the analyses investigating potential causal relationships, we plan to construct directed acyclic graphs (DAGs) using the program dagitty (http://www.dagitty.net/). The use of DAGs is becoming quite common in the nutrition field due to their ability to portray the assumptions about the relationships between the variables in the causal structures and establishing the minimal set of variables sufficient for adjusting that blocks all backdoor paths and does not open any closed pathways (53). The DAGs are particularly useful to identify the minimal set of confounders for adjustment in the regression models. In the cases where we plan to have the analyses stratified (according to socioeconomic conditions, for example), a DAG will be constructed for each subgroup. The consideration of potential confounders will be informed by the existing literature and substantive knowledge of the investigators. We also plan to apply sensitivity analyses to consider the impact of unmeasured confounding (54).

In the studies aiming to test associations between maternal and child characteristics, it is crucial to deal with missing data in the variables included in the models as exposure and confounders. Hence, we plan to consider the mechanism underlying the occurrence of missing data (whether missingness occurred completely at random – MCAR, at random – MAR, or not at random - MNAR), the pattern of

For peer review only - http://bmjopen.bmj.com/site/about/guidelines.xhtml

For peer review only

missingness (monotonic or non-monotonic), and the most appropriate method to deal with each of those. Techniques such as multiple imputation, inverse probability weighting, and full-information maximum likelihood will be considered (55, 56). We also plan to follow the recently published 'Framework for the treatment and reporting of missing data in observational studies' (TARMOS framework) to deal with missing data in a study-based approach (57). All the proposed analyses will be performed in R or Stata.

Patient and public involvement

Patients and the public were not involved in any step of this study.

ETHICS AND DISSEMINATION

The objectives described in this protocol were approved by the Research Ethics Committees from the Gonçalo Moniz Institute/Oswaldo Cruz Foundation (CAAE: 56003716.0.0000.0040), the Institute of Collective Health/Federal University of Bahia (CAAE: 41695415.0.0000.5030), the Federal University of Rio de Janeiro (CAAE: 85914318.2.0000.5275; 18447919.3.0000.5264), and the Federal University of Minas Gerais (CAAE: 37534620.3.0000.5149). To ensure data privacy and confidentiality, all data linkage procedures will be conducted in a physically and virtually secure environment and follow strict internal information security measures (26). Access to linked and de-identified datasets will be granted to authorized researchers only. The analysis will be performed in the CIDACS data analysis environment, a safe virtual infrastructure that provides remote data access and analysis tools. The final linked and de-identified datasets will receive a Digital Object Identifier (DOI), and the complete specification of how the dataset was created will be made available upon request.

For peer review only - http://bmjopen.bmj.com/site/about/guidelines.xhtml

Manuscripts from studies of this protocol will be submitted to open-access journals. In addition, we will promote stakeholder events and produce policy briefs to disseminate the results to policymakers and the broader public.

AUTHOR CONTRIBUTION

TRBC and NJS contributed equally to this paper and share the first authorship. GK, RCRS and MLB share the senior authorship. TRBC, NJS, and GK wrote the manuscript with input from ESP, IRF, RFL, LCR, SVK, AHL, RD, AP, GVM, RCRS, and MLB. GK, GVM, and RCRS are the main responsible for the design of the research. RCRS is the coordinator of the GeNut-CIDACS group. MLB is the coordinator of the CIDACS and the responsible to grant access to the datasets. The authors from the GeNut-CIDACS group contributed to the revision of the manuscript. TRBC, NJS, and GK had primary responsibility for the final content. All authors read and approved the final manuscript.

FUNDING STATEMENT

This study is funded by the Brazilian Ministry of Health (TED 64/2022, process 25000.148278/2022-10). CIDACS receives core support from the Wellcome Trust (202912/Z/16/Z), the Brazilian Health Surveillance Secretariat, the Brazilian Ministry of Health, the State of Bahia, the Research Support Foundation of the State of Bahia (FAPESB), the Research and Project Funding Agency (FINEP), and the Secretariat of Science and Technology of the State of Bahia (SECTI). SVK acknowledges funding from a NRS Senior Clinical Fellowship (SCAF/15/02), the Medical Research Council (MC_UU_00022/2), and the Scottish Government Chief Scientist Office (SPHSU17).

For peer review only - http://bmjopen.bmj.com/site/about/guidelines.xhtml

NJS acknowledges support from the Spanish Ministry of Science and Innovation and State Research Agency through the "Centro de Excelencia Severo Ochoa 2019-2023" Program (CEX2018-000806-S) and support from the Generalitat de Catalunya through the CERCA Program.

COMPETING INTERESTS

The authors declare that they have no competing interests.

DATA SHARING STATEMENT

Not applicable.

For peer review only

For peer review only - http://bmjopen.bmj.com/site/about/guidelines.xhtml

REFERENCES

1. Aune D, Saugstad OD, Henriksen T, Tonstad S. Maternal body mass index and the risk of fetal death, stillbirth, and infant death: a systematic review and meta-analysis. JAMA 2014;311(15):1536-46.

2. Adane AA, Shepherd CCJ, Lim FJ, et al. The impact of pre-pregnancy body mass index and gestational weight gain on placental abruption risk: a systematic review and meta-analysis. Arch Gynecol Obstet 2019;300(5):1201-10.

3. Goldstein RF, Abell SK, Ranasinha S, et al. Association of gestational weight gain with maternal and infant outcomes: a systematic review and meta-analysis. JAMA 2017;317(21):2207-25.

4. Saucedo M, Esteves-Pereira AP, Pencolé L, et al. Understanding maternal mortality in women with obesity and the role of care they receive: a national case-control study. Int J Obes 2021;45(1):258-65.

5. Gaillard R, Steegers EA, Franco OH, et al. Maternal weight gain in different periods of pregnancy and childhood cardio-metabolic outcomes. The Generation R Study. Int J Obes 2015;39(4):677-85.

6. Gaillard R, Welten M, Oddy WH, et al. Associations of maternal prepregnancy body mass index and gestational weight gain with cardio-metabolic risk factors in adolescent offspring: a prospective cohort study. BJOG 2016;123(2):207-16.

7. Voerman E, Santos S, Inskip H, et al. Association of gestational weight gain with adverse maternal and infant outcomes. JAMA 2019;321(17):1702-15.

8. Voerman E, Santos S, Patro Golab B, et al. Maternal body mass index, gestational weight gain, and the risk of overweight and obesity across childhood: An individual participant data meta-analysis. PLoS Med 2019;16(2).

For peer review only - http://bmjopen.bmj.com/site/about/guidelines.xhtml

9. Ehrlich SF, Hedderson MM, Feng J, et al. Change in body mass index between pregnancies and the risk of gestational diabetes in a second pregnancy. Obstet Gynecol 2011;117(6):1323-30.

10. Brunner S, Stecher L, Ziebarth S, et al. Excessive gestational weight gain prior to glucose screening and the risk of gestational diabetes: a meta-analysis. Diabetologia 2015;58(10):2229-37.

11. Oteng-Ntim E, Mononen S, Sawicki O, Seed P, Bick D, Poston L. Interpregnancy weight change and adverse pregnancy outcomes: A systematic review and meta-analysis. BMJ Open 2018;8:e018778.

12. Rogozinska E, Zamora J, Marlin N, et al. Gestational weight gain outside the Institute of Medicine recommendations and adverse pregnancy outcomes: analysis using individual participant data from randomised trials. BMC Pregnancy Childbirth 2019;19(1):322.

13. Teulings NEWD, Masconi KL, Ozanne SE, et al. Effect of interpregnancy weight change on perinatal outcomes: systematic review and meta-analysis. BMC Pregnancy Childbirth 2019;19(1):386.

14. Poston L, Caleyachetty R, Cnattingius S, et al. Preconceptional and maternal obesity: epidemiology and health consequences. Lancet Diabetes Endocrinol 2016;4(12):1025-36.

15. IBGE. Instituto Brasileiro de Geografia e Estatística. Pesquisa nacional de saúde 2019: atenção primária à saúde e informações antropométricas. Rio de Janeiro: IBGE; 2020.

16. Taylor EJ, Wilding S, Ziauddeen N, et al. Change in modifiable maternal characteristics and behaviours between consecutive pregnancies and offspring adiposity: A systematic review. Obes Rev 2020;21(11):e13048.

For peer review only - http://bmjopen.bmj.com/site/about/guidelines.xhtml

17. Wallace JM, Bhattacharya S, Horgan GW. Weight change across the start of three consecutive pregnancies and the risk of maternal morbidity and SGA birth at the second and third pregnancy. PLoS One 2017;12(6):e0179589.

18. Bjerregaard AA, Halldorsson TI, Tetens I, Olsen SF. Mother's dietary quality during pregnancy and offspring's dietary quality in adolescence: Follow-up from a national birth cohort study of 19,582 mother-offspring pairs. PLoS Med 2019;16(9):e1002911.

19. Durão C, Severo M, Oliveira A, et al. Association of maternal characteristics and behaviours with 4-year-old children's dietary patterns. Matern Child Nutr 2017;13(2).

20. Daymont C, Ross ME, Russell Localio A, et al. Automated identification of implausible values in growth data from pediatric electronic health records. J Am Med Inform Assoc 2017;24(6):1080-7.

21. Murdoch TB, Detsky AS. The inevitable application of big data to health care. JAMA 2013;309(13):1351-2.

22. Mooney SJ, Westreich DJ, El-Sayed AM. Commentary: Epidemiology in the era of big data. Epidemiology. 2015;26(3):390-4.

23. Harron K, Dibben C, Boyd J, et al. Challenges in administrative data linkage for research. Big Data Soc 2017;4(2):2053951717745678.

24. Victora CG, Barros FC. Cohorts in low- and middle-income countries: from still photographs to full-length movies. J Adolesc Health 2012;51(6 Suppl):S3-4.

25. Benchimol EI, Smeeth L, Guttmann A, et al. The REporting of studies Conducted using Observational Routinely-collected health Data (RECORD) statement. PLoS Med 2015;12(10):e1001885.

26. Barreto ML, Rodrigues L, Denaxas S, et al. The Center for Data and Knowledge Integration for Health (CIDACS): an experience of linking health and social data in Brazil. Int J Popul Data Sci 2019;4(2).

For peer review only - http://bmjopen.bmj.com/site/about/guidelines.xhtml

27. Pescarini JM, Craig P, Allik M, et al. Evaluating the impact of the Bolsa Familia conditional cash transfer program on premature cardiovascular and all-cause mortality using the 100 million Brazilian cohort: a natural experiment study protocol. BMJ Open 2020;10(11):e039658.

28. Paixao ES, Cardim LL, Falcao IR, et al. Cohort Profile: Centro de Integracao de Dados e Conhecimentos para Saude (CIDACS) Birth Cohort. Int J Epidemiol 2021;50(1):37-8.

29. Szwarcwald CL, Leal MDC, Esteves-Pereira AP, et al. [Evaluation of data from the Brazilian Information System on Live Births (SINASC)]. Cad Saude Publica 2019;35(10):e00214918.

30. Jorge MHPdM, Laurenti R, Gotlieb SLD. Análise da qualidade das estatísticas vitais brasileiras: a experiência de implantação do SIM e do SINASC. Cien Saude Colet 2007;12:643-54.

31. Brasil. Ministério da Saúde. Secretaria de Atenção à Saúde. Departamento de Atenção Básica. Política Nacional de Alimentação e Nutrição. In: Ministério da Saúde. Secretaria de Atenção à Saúde. Departamento de Atenção Básica. Brasília: Ministério da Saúde; 2013. p. 84.

32. Ali MS, Ichihara MY, Lopes LC, et al. Administrative Data Linkage in Brazil: Potentials for Health Technology Assessment. Front Pharmacol 2019;10:984.

33. Barbosa GCG, Ali MS, Araujo B, et al. CIDACS-RL: a novel indexing search and scoring-based record linkage system for huge datasets with high accuracy and scalability. BMC Med Inform Decis Mak 2020;20(1):289.

34. Almeida D, Gorender D, Ichihara MY, et al. Examining the quality of record linkage process using nationwide Brazilian administrative databases to build a large birth cohort. BMC Med Inform Decis Mak 2020;20(1):173.

For peer review only - http://bmjopen.bmj.com/site/about/guidelines.xhtml

35. Yang S, Hutcheon JA. Identifying outliers and implausible values in growth trajectory data. Ann Epidemiol 2016;26(1):77-80 e1-2.

36. Shi J, Korsiak J, Roth DE. New approach for the identification of implausible values and outliers in longitudinal childhood anthropometric data. Ann Epidemiol 2018;28(3):204-11 e3.

37. Welch C, Petersen I, Walters K, et al. Two-stage method to remove population- and individual-level outliers from longitudinal data in a primary care database. Pharmacoepidemiol Drug Saf 2012;21(7):725-32.

38. Boone-Heinonen J, Tillotson CJ, O'Malley JP, et al. Not so implausible: impact of longitudinal assessment of implausible anthropometric measures on obesity prevalence and weight change in children and adolescents. Ann Epidemiol 2019;31:69-74 e5.

39. Carrilho TRB, Farias DR, Batalha MA, et al. Brazilian Maternal and Child Nutrition Consortium: establishment, data harmonization and basic characteristics. Sci Rep 2020;10(1):14869.

40. WHO Multicentre Growth Reference Study Group. Assessment of differences in linear growth among populations in the WHO Multicentre Growth Reference Study. Acta Paediatr Suppl 2006;95(S450):56-65.

41. Jackson JW, VanderWeele TJ. Intersectional decomposition analysis with differential exposure, effects, and construct. Soc Sci Med 2019;226:254-9.

42. Leonard SA, Rasmussen KM, King JC, Abrams B. Trajectories of maternal weight from before pregnancy through postpartum and associations with childhood obesity. Am J Clin Nutr 2017;106(5):1295-301.

43. Buuren Sv. Broken stick model for irregular longitudinal data. J Stat Softw. 2021.

44. Anderson C, Hafen R, Sofrygin O, et al. Comparing predictive abilities of longitudinal child growth models. Stat Med 2019;38(19):3555-70.

For peer review only

45. Cole TJ, Donaldson MD, Ben-Shlomo Y. SITAR--a useful instrument for growth curve analysis. Int J Epidemiol 2010;39(6):1558-66.

46. Riddell CA, Platt RW, Bodnar LM, Hutcheon JA. Classifying gestational weight gain trajectories using the SITAR growth model. Paediatr Perinat Epidemiol 2017;31(2):116-25.

47. Rumelhart DE, Hinton GE, Williams RJ. Learning representations by back-propagating errors. Nature 1986;323(6088):533-6.

48. Jang JR. ANFIS: adaptive-network-based fuzzy inference system. IEEE Trans Syst Man Cybern 1993;23(3):665-85.

49. Chen YH, Ferguson KK, Meeker JD, et al. Statistical methods for modeling repeated measures of maternal environmental exposure biomarkers during pregnancy in association with preterm birth. Environ Health 2015;14:9.

50. Harrell FE, Jr., Lee KL, Pollock BG. Regression models in clinical studies: determining relationships between predictors and response. J Natl Cancer Inst 1988;80(15):1198-202.

51. Sauerbrei W, Royston P. Building multivariable prognostic and diagnostic models: transformation of the predictors by using fractional polynomials. J R Stat Soc Series A 1999;162(1):71-94.

52. Royston P, Ambler G, Sauerbrei W. The use of fractional polynomials to model continuous risk variables in epidemiology. Int J Epidemiol 1999;28(5):964-74.

53. Greenland S, Pearl J, Robins JM. Causal diagrams for epidemiologic research. Epidemiology 1999;10(1):37-48.

54. VanderWeele TJ, Ding P. Sensitivity analysis in observational research: Introducing the E-Value. Ann Intern Med 2017;167(4):268-74.

For peer review only - http://bmjopen.bmj.com/site/about/guidelines.xhtml

55. Little TD, Jorgensen TD, Lang KM, Moore EW. On the joys of missing data. J Pediatr Psychol 2014;39(2):151-62.

56. Huque MH, Carlin JB, Simpson JA, Lee KJ. A comparison of multiple imputation methods for missing data in longitudinal studies. BMC Med Res Methodol 2018;18(1):168.

57. Lee KJ, Tilling KM, Cornish RP, et al. Framework for the treatment and reporting of missing data in observational studies: The Treatment And Reporting of Missing data in Observational Studies framework. J Clin Epidemiol 2021;134:79-88.

58. Lima EE, Queiroz BL. Evolution of the deaths registry system in Brazil: associations with changes in the mortality profile, under-registration of death counts, and ill-defined causes of death. Cad Saude Publica 2014;30(8):1721-30.

59. Carrilho TRB, Rasmussen KM, Farias DR, et al. Agreement between self-reported pre-pregnancy weight and measured first-trimester weight in Brazilian women. BMC Pregnancy Childbirth. 2020;20(1):734.

60. WHO Expert Committee on Physical Status. Physical status: the use and interpretation of anthropometry: report of a WHO Expert Committee. Geneva: World Health Organization; 1995. x, 452 p. p.

61. Pole JD, Dodds LA. Maternal outcomes associated with weight change between pregnancies. Can J Public Health 1999;90(4):233-6.

62. Oteng-Ntim E, Mononen S, Sawicki O, et al. Interpregnancy weight change and adverse pregnancy outcomes: a systematic review and meta-analysis. BMJ Open 2018;8(6):e018778.

63. Martinez-Hortelano JA, Berlanga-Macias C, Pozuelo-Carrascosa DP, et al. Interpregnancy weight change and perinatal outcomes: A protocol for a systematic review and meta-analysis. Medicine 2019;98(20):e15470.

For peer review only - http://bmjopen.bmj.com/site/about/guidelines.xhtml

64. Amorim AR, Linne Y, Kac G, Lourenco PM. Assessment of weight changes during and after pregnancy: practical approaches. Matern Child Nutr 2008;4(1):1-13.

65. Kac G, Carrilho TRB, Rasmussen KM, et al. Gestational weight gain charts: results from the Brazilian Maternal and Child Nutrition Consortium. Am J Clin Nutr 2021;113(5):1351-60.

66. World Health Organization. ICD-10: International Statistical Classification of Diseases and Related Health Problems, 10th Revision. Geneva: WHO; 2010.

67. Villar J, Cheikh Ismail L, Victora CG, et al. International standards for newborn weight, length, and head circumference by gestational age and sex: the Newborn Cross-Sectional Study of the INTERGROWTH-21st Project. Lancet 2014;384(9946):857-68.

68. World Health Organization. WHO child growth standards: length/height-for-age, weight-for-age, weight-for-length, weight-for-height and body mass index-for-age: methods and development. Geneva: World Health Organization; 2006.

69. Brasil. Ministério da Saúde. Orientações para a coleta e análise de dados antropométricos em serviços de saúde. Norma Técnica do Sistema de Vigilância Alimentar e Nutricional - SISVAN. Brasília: Ministério da Saúde; 2011.

70. Monteiro PO, Victora CG. Rapid growth in infancy and childhood and obesity in later life - a systematic review. Obes Rev 2005;6(2):143-54.

For peer review only

For peer review only - http://bmjopen.bmj.com/site/about/guidelines.xhtml

TABLES

Table 1. Variables available in each data source to be used in the maternal and child nutrition research program.

Database	Data collection process	Available variables	Coverage of the database	Period available
100 Million Brazilian Cohort baseline	The baseline of the 100 million Brazilian cohort was created using administrative records from individuals ≥ 16 years of age whose families applied for social assistance via the *Cadastro Único para Programas Sociais*.	Characteristics of the registered individuals (year of application/entry, gender, age at entry year, self-declared race/ethnicity, education, marital status, head of the family), characteristics of the household (municipality of residence, urban/rural area of residence, household building material, water supply, sewage disposal, waste disposal/garbage collection, electricity, number of individuals in the household, number of rooms in the household); 'Bolsa Familia*' information (identification of beneficiary families or not, receipt start date, final receipt date, receipt time in days).	More than 50% of the Brazilian population	2001 - 2018
SINASC (Live Births Information System)	The form (declaration of live birth) is completed by a health professional present at the delivery, and the system records information about all live births in Brazil.	Characteristics of the newborn (sex; Apgar score in the 1st and 5th minute, birth weight, presence of abnormalities, congenital anomalies identified at birth using the ICD-10 code), place of birth, characteristics of the mother (name, age, marital status, education, race, place of residence), father's age, characteristics of pregnancy and delivery (number of previous pregnancies of live births, stillbirth or abortion, length of gestation, type of delivery, number of fetuses, number of visits to prenatal care facilities, month of pregnancy in which prenatal care started, which health professionals were present at the delivery).	Approximately 97% of the Brazilian live births	2001 – 2018

For peer review only - http://bmjopen.bmj.com/site/about/guidelines.xhtml

SIM (Mortality Information System)	The form (declaration of death) is completed by the physician who attests the cause of death. The system records all deaths in Brazil, and it differentiates between fetal and non-fetal deaths.	Characteristics of the dead person (date of death and birth, name, name of the mother and father, sex, race, marital status, occupation and education, address), identification of the place of death (hospital, home, public place and includes the address of the place), characteristics of the mother (name, age, marital status, education, occupation, race, number of births, place of residence, length of gestation, number of previous stillbirths or abortions, type of delivery, number of fetus in the current pregnancy – filled in case of fetal death or infant mortality), birth weight (filled only in case of fetal deaths or infant mortality), and cause of death using ICD-10 code.	SIM coverage has been improving over time, reaching around 95% in some regions**.	2011 – 2018
SISVAN (Food and Nutrition Surveillance System)	The forms are completed by primary care workers in the public healthcare system. Anthropometric and food intake markers data of the Brazilian Unified Health System (SUS) users are collected.	Date of birth, age; sex, race/ethnicity, education, participation in social/health programs, diseases, and intercurrences, food intake data (food markers according to each stage of the lifecycle), and anthropometric data: weight and height, birth weight for children under two years, waist circumference for adults, calf circumference for elderly, and pre-pregnancy weight for pregnant women;	About 30% among under-five children and 17% among pregnant women	2008 - 2017

*Bolsa Familia is the conditional cash transfer program implemented in Brazil since 2004. ** In 2010, the system's coverage was estimated to be 95% in some Brazilian regions (58).

For peer review only - http://bmjopen.bmj.com/site/about/guidelines.xhtml

Table 2. Main variables to be used in the maternal and child nutrition research program.

Variable	Characteristics
Maternal variables	
Mode of delivery	• It is collected as vaginal or cesarean.<br>• Since 2011, there is also the information if the labor was induced.
Maternal pre-pregnancy BMI	• It is calculated based on weight (kg) and height (m) using the formula: BMI weight (kg)/height $(m)^2$.<br>• Self-reported pre-pregnancy weight will be used for its calculation, considering the good agreement observed between self-reported and measured first-trimester weight in a previous study using the SISVAN data (59).<br>• Continuous variable (kg/m^2).<br>• The WHO cutoffs will be used: underweight (< 18.5 kg/m^2), normal weight (≥ 18.5 and < 25.0 kg/m^2), overweight (≥ 25.0 and < 30.0 kg/m^2) and obesity (≥ 30.0 kg/m^2) (60).
Maternal weight changes between pregnancies	• It is calculated as the difference in self-reported pre-pregnancy weights between consecutive pregnancies.<br>• Continuous variable (kg).<br>• Percentage of weight change (61).
Maternal BMI change between pregnancies	• It is calculated as the difference between BMIs at the beginning of consecutive pregnancies.<br>• It will be used as a continuous variable (units of increase/decrease in BMI) or categorized (62).<br>• The change in BMI category (based on the WHO cutoffs) between pregnancies will also be used (63).
Gestational weight gain (GWG)	• Several ways to calculate GWG will be tested in this study.<br>• Total GWG is calculated as the difference between the weight measured up to 14 days before delivery and self-reported pre-pregnancy weight (64).<br>• Cumulative GWG is calculated as the difference between GWG measured in prenatal visits and self-reported pre-pregnancy weight (64).<br>• Continuous variable (kg).<br>• Z scores of the Brazilian GWG charts (65).<br>• GWG per trimester can also be considered.
Postpartum weight retention	• It is calculated as the difference between the weight measured at 6 and 12 months postpartum and self-reported pre-pregnancy weight.<br>• Continuous (kg) variable.<br>• Categorical variable – we plan to test several cutoffs for its classification.
Maternal weight trajectories	• This outcome will be created in objective 1, and the groups of trajectories identified will be used in the following studies.
Food intake markers	• The following markers are available: consumption of beans; fresh fruits; vegetables; hamburgers or processed, mixed meat products; sugar-sweetened beverages; instant noodles, packaged snacks, or crackers; stuffed biscuits/cookies or candies.<br>• The response options are 'yes/no', and the time frame always refers to the intake on the day before the visit. These markers are collected before, during, and after pregnancy.
Maternal mortality	• Maternal death will be defined as the death of women during pregnancy or up to 42 days after delivery, due to any cause related to or aggravated by the pregnancy, but not due to accidental or incidental causes (66).
Child variables	
Gestational age at birth	• This information is available in weeks since 2011. Before, it is available only categorized as <22 weeks, 22 to 27 weeks; 28 to 31 weeks; 32 to 36 weeks; 37 to 41 weeks; and 42 weeks or more.

For peer review only - http://bmjopen.bmj.com/site/about/guidelines.xhtml

	• It can be used in weeks or classified as preterm (< 37 weeks)/term birth (≥ 37 weeks) (66).<br>• Preterm birth can also be divided into extremely preterm (<28 weeks), very preterm (28–<32 weeks), and moderate or late preterm (32–<37 completed weeks)(66).
Birth weight	• Continuous, in grams<br>• Classified as large (> P90) and small (< P10) for gestational age, according to Intergrowth-21st sex and gestational age-specific charts (67).<br>• Classified as low birth weight (LBW, < 2500g) and macrosomia (>4000g) (68).<br>• LBW can also be classified into very LBW (<1500g) and extremely LBW (< 1000g)(66).
BMI up to 5 years old	• It is calculated based on measured weight (kg) and height (m).<br>• Continuous variable (kg/m^2).<br>• BMI-for-age z-scores for sex according to the WHO charts (68) will be used.<br>• It can also be used categorized as proposed by the Brazilian Ministry of Health (69): Underweight (BMI-for-age<-2 z score); Normal (BMI-for-age≥-2 and ≤ 2 z score); Overweight (BMI-for-age>2 and ≤ 3 z score); Obesity (BMI-for-age>3 z score).
Rapid infant growth	• It is determined based on weight-for-age z scores according to the WHO charts (68).<br>• It will be classified when the z scores in weight-for-age between two different ages up to 5 years old is > 0.67 (70).
Growth trajectories	• This outcome will be created in objective 2, and the groups of trajectories identified will be used in the following studies.
Food intake markers	The following markers are collected:<br>• For children < 6 months old: consumption of breast milk; porridge; water/ tea; cow milk; infant formula; fruit juice; fruits; salt food; other food/ beverages.<br>• For children 6 – 23 months old: consumption of breast milk; whole fruit in pieces or mashed (and the daily frequency); salt food (and the daily frequency and how the food was offered - in pieces/ kneaded/ passed through a sieve/ blended/ only the broth); milk other than breast milk; milk porridge; yogurt; vegetables; orange-colored vegetables (carrots, pumpkin, papaya) or fruit or dark green leaves (spinach, kale); leaf greenery (lettuce, cabbage); meat (beef, chicken, pork, fish) or eggs; liver; beans; rice, potatoes, yams, cassava, cassava flour, or pasta; hamburger and/or sausages; sugar-sweetened beverages; instant noodles, packaged snacks, or crackers; stuffed biscuits/cookies or candies.<br>• For children > 24 months old: consumption of beans; fresh fruits; vegetables; hamburgers or processed, mixed meat products; sugar-sweetened beverages; instant noodles, packaged snacks, or crackers; stuffed biscuits/cookies or candies.<br>• The response options are 'yes/no', and the time frame always refers to the intake on the day before the visit.
Child mortality	• Child mortality refers to the death of children under five years old (66).<br>• It is also possible to work with perinatal, neonatal, and postneonatal mortality.<br>• Perinatal mortality includes deaths in the first week of life and fetal deaths (stillbirths) (66).<br>• Neonatal mortality refers to deaths that occurred up to 28 days after birth. It can be divided into two components: a. early neonatal mortality, which includes the deaths among live births in the first 6 days of life; b. late neonatal mortality includes deaths among live births between 7 and 27 days of life (66).<br>• Post neonatal mortality refers to the deaths among live births between 28 and 364 completed days of life (66).

Notes: BMI: Body mass index; GWG: Gestational weight gain; SISVAN: Food and Nutrition Surveillance System; WHO: World Health Organization.

For peer review only - http://bmjopen.bmj.com/site/about/guidelines.xhtml

Table 3. Summary of objectives, outcomes, and proposed analytic approaches.

Objectives	Outcomes/main variables	Analytical approaches
1. Describe maternal body weight trajectories during the life course (adolescence, adulthood, pregnancy, and postpartum).	Maternal weight, age, and gestational age/number of postpartum days in each visit.	• Latent-class growth models<br>• Broken stick models<br>• SITAR models
2. Describe child weight, height, and BMI trajectories until the age of five.	Children's weight, height, BMI (crude and z scores), age in each measurement.	• Latent-class growth models<br>• Broken stick models<br>• SITAR models
3. Create and validate models to predict childhood obesity at the age of five.	*Predictors:* A set of predictors will be considered for the models – characteristics of the child, characteristics of the mother, and family/environment factors.<br><br>*Outcomes:* childhood obesity at the age of five and rapid infant growth at different ages (12, 18, 24, 36, 48, and 59 months).	Machine learning models using:<br>• Artificial Neural Networks<br>• Adaptive Fuzzy Systems
4. Estimate the effect of pre-pregnancy BMI, GWG, maternal weight, BMI changes between pregnancies, and maternal weight trajectories on adverse maternal and neonatal outcomes and child growth trajectories.	*Exposures:* Pre-pregnancy BMI, GWG, weight, and BMI changes.<br><br>*Outcomes:* LGA, SGA, LBW, macrosomia, preterm birth, mode of delivery, child BMI and growth indicators, child growth trajectories defined in goal 2, rapid infant growth, child mortality, and maternal mortality.	• Generalized linear models (Logistic and Poisson regression for categorical and linear and quantile regression for continuous outcomes)<br>• Linear mixed-effects models for longitudinal outcomes<br>• Confounding will be considered using directed acyclic graphs
5. Estimate the effect of pre-pregnancy BMI, GWG, and maternal weight and BMI changes on adverse maternal and neonatal outcomes in the subsequent pregnancy	*Exposures:* Pre-pregnancy BMI, weight, and BMI changes.<br><br>*Outcomes:* LGA, SGA, LBW, macrosomia, preterm birth, mode of delivery, children's BMI and growth indicators, growth trajectories defined in goal 2, rapid infant growth, neonatal and child mortality, and maternal mortality in the consecutive pregnancy.	• Generalized linear models (Logistic and Poisson regression for categorical and linear regression for continuous outcomes)<br>• Linear mixed-effects models for longitudinal outcomes<br>• Confounding will be considered using directed acyclic graphs
6. Estimate the effect of maternal and infant feeding practices on child nutritional status and growth trajectories.	*Exposures:* Food intake markers during pregnancy, breastfeeding, and complementary feeding.<br><br>*Outcomes:* child BMI and growth indicators, growth trajectories defined in goal 2, and rapid infant growth.	• Generalized linear models (Logistic and Poisson regression for categorical and linear regression for continuous outcomes)<br>• Linear mixed-effects models for longitudinal outcomes<br>• Confounding will be considered using directed acyclic graphs

Note: BMI: Body mass index; GWG: Gestational weight gain; SITAR: Super-Imposition by Translation and Rotation; WHO: World Health Organization.

For peer review only - http://bmjopen.bmj.com/site/about/guidelines.xhtml

FIGURE LEGENDS

Figure 1. Theoretical framework guiding the investigation of maternal and child nutrition within the 100 million Brazilian cohort.

For peer review only

For peer review only - http://bmjopen.bmj.com/site/about/guidelines.xhtml

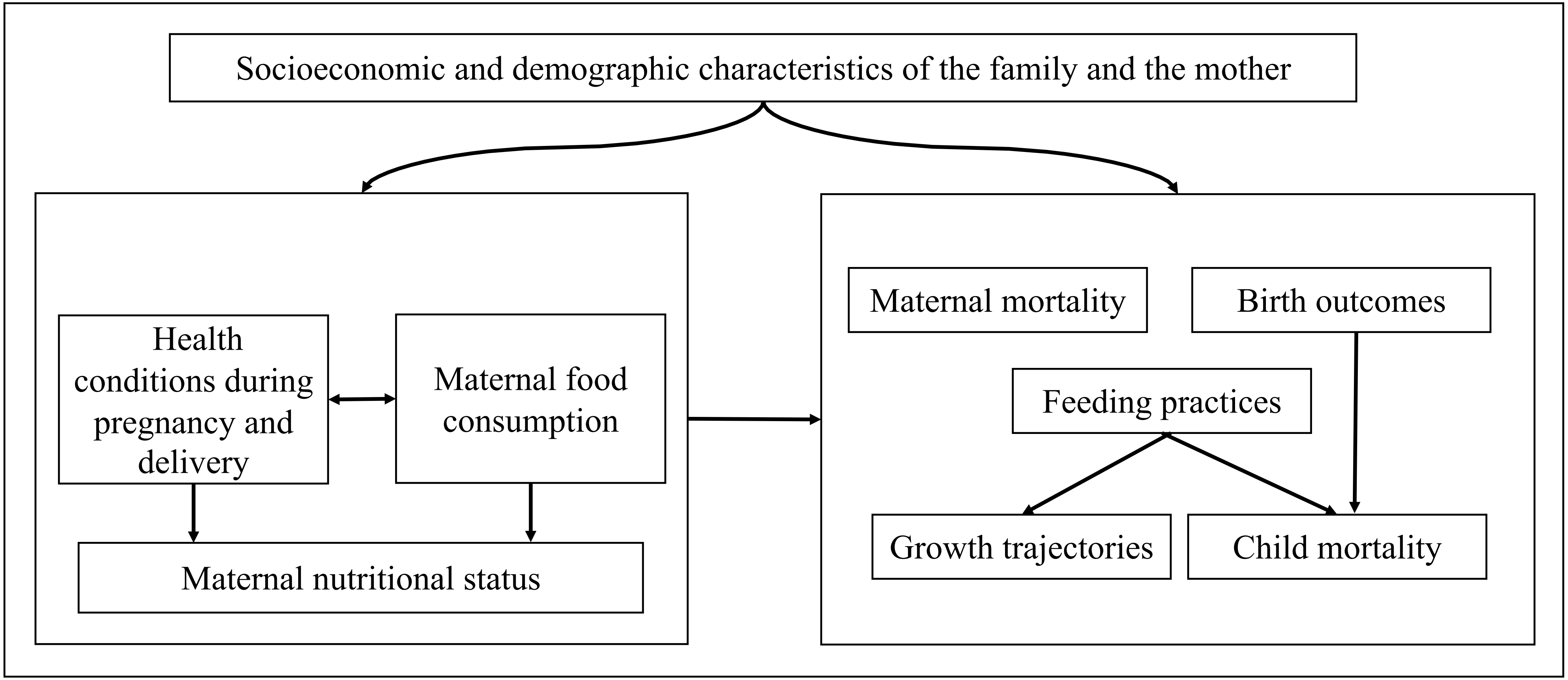
Socioeconomic and demographic characteristics of the family and the mother
Health conditions during pregnancy and delivery
Maternal food consumption
Maternal nutritional status
Maternal mortality
Birth outcomes
Feeding practices
Growth trajectories
Child mortality

BMJ Open

Maternal and child nutrition program of investigation within the 100 million Brazilian Cohort: study protocol

Journal:	*BMJ Open*
Manuscript ID	bmjopen-2023-073479.R1
Article Type:	Protocol
Date Submitted by the Author:	11-Jul-2023
Complete List of Authors:	Carrilho, Thais; Federal University of Rio de Janeiro, Nutritional Epidemiology Observatory, Josué de Castro Institute of Nutrition<br>Silva, Natanael; Oswaldo Cruz Foundation, Centre for Data and Knowledge Integration for Health, Gonçalo Moniz Institute; University of Barcelona, Barcelona Institute for Global Health, Hospital Clínic<br>Paixão, Enny; London School of Hygiene & Tropical Medicine, Epidemiology and Population Health; Oswaldo Cruz Foundation, Centre for Data and Knowledge Integration for Health, Gonçalo Moniz Institute<br>Falcão, Ila; Oswaldo Cruz Foundation, Centre for Data and Knowledge Integration for Health, Gonçalo Moniz Institute; Federal University of Bahia, School of Nutrition<br>Fiaccone, Rosemeire; Federal University of Bahia, Institute of Mathematics and Statistics; Oswaldo Cruz Foundation, Centre for Data and Knowledge Integration for Health, Gonçalo Moniz Institute<br>Rodrigues, Laura; London School of Hygiene & Tropical Medicine, Department of Infectious Disease Epidemiology; Oswaldo Cruz Foundation, Centre for Data and Knowledge Integration for Health, Gonçalo Moniz Institute<br>Katikireddi, Srinivasa; University of Glasgow, MRC/CSO Social & Public Health Sciences Unit<br>Leyland, Alastair; University of Glasgow, MRC/CSO Social & Public Health Sciences Unit<br>Dundas, Ruth; University of Glasgow, MRC/CSO Social and Public Health Sciences Unit<br>Pearce, Anna; University of Glasgow, MRC/CSO Social & Public Health Sciences Unit<br>Velasquez-Melendez, Gustavo; Federal University of Minas Gerais, Department of Maternal and Child Nursing and Public Health, Nursing School<br>Kac, Gilberto; Federal University of Rio de Janeiro, Nutritional Epidemiology Observatory, Josué de Castro Nutrition Institute<br>Silva, Rita de Cássia; Oswaldo Cruz Foundation, Centre for Data and Knowledge Integration for Health, Gonçalo Moniz Institute; Federal University of Bahia, School of Nutrition<br>Barreto, Mauricio; Oswaldo Cruz Foundation, Centre for Data and Knowledge Integration for Health, Gonçalo Moniz Institute; Federal University of Bahia, Institute of Collective Health<br>Centre for Data and Knowledge Integration for Health, Nutrition Study Group (GeNut-CIDACS); Oswaldo Cruz Foundation, Centre for Data and Knowledge Integration for Health, Gonçalo Moniz Institute

**Primary Subject Heading**:	Epidemiology
Secondary Subject Heading:	Public health, Nutrition and metabolism
Keywords:	NUTRITION & DIETETICS, Community child health < PAEDIATRICS, PUBLIC HEALTH

Maternal and child nutrition program of investigation within the 100 million Brazilian Cohort: study protocol

Thais Rangel Bousquet Carrilho[1], Natanael de Jesus Silva[2,3], Enny S. Paixão[2,4], Ila Rocha Falcão[2,5], Rosemeire L. Fiaccone[2,6], Laura C. Rodrigues[2,4], Srinivasa Vittal Katikireddi[7], Alastair H. Leyland[7], Ruth Dundas[7], Anna Pearce[7], Gustavo Velasquez-Melendez[8], Gilberto Kac[1], Rita de Cássia Ribeiro-Silva[2,5*], Mauricio L. Barreto[2,9], on behalf of the Nutrition Study Group of the Center for Data and Knowledge Integration for Health (GeNut-CIDACS)

1. Nutritional Epidemiology Observatory, Josué de Castro Nutrition Institute, Federal University of Rio de Janeiro, Rio de Janeiro, RJ, Brazil.

2. Centre for Data and Knowledge Integration for Health, Gonçalo Moniz Institute, Oswaldo Cruz Foundation, Salvador, BA, Brazil.

3. Barcelona Institute for Global Health, Hospital Clínic, University of Barcelona, Barcelona, Spain.

4. Epidemiology and Population Health, London School of Hygiene and Tropical Medicine, London, UK.

5. School of Nutrition, Federal University of Bahia, Salvador, BA, Brazil.

6. Institute of Mathematics and Statistics, Federal University of Bahia, Salvador, BA, Brazil.

7. MRC/CSO Social & Public Health Sciences Unit, University of Glasgow School of Life Sciences, Glasgow, UK.

8. Department of Maternal and Child Nursing and Public Health, Nursing School, Federal University of Minas Gerais, Belo Horizonte, MG, Brazil.

For peer review only - http://bmjopen.bmj.com/site/about/guidelines.xhtml

9. Institute of Collective Health, Federal University of Bahia, Salvador, BA, Brazil.

Corresponding author:

Rita de Cássia Ribeiro-Silva. School of Nutrition, Federal University of Bahia. Av. Araújo Pinho, nº 32, Canela – Salvador, Bahia, Brazil / CEP: 40.110-150. Email: rcrsilva@ufba.br.

Collaborators of GeNut CIDACS:

The following are members of the GeNut-CIDACS – Nutrition Study Group of the Center for Data and Knowledge Integration for Health: Alastair H Leyland, Aline S Rocha, Anna Pearce, André A Mendes, Andrêa JF Ferreira, Audêncio Victor, Camila SS Teixeira, Carolina S Vieira, Craig Anderson, Dayana R Farias, Davide Rasella, Eduardo AF Nilson, Elizabete J Pinto, Enny S Paixão, Flávia JO Alves, Giesy R Souza, Gilberto Kac, Gustavo Velasquez-Melendez, Ila R Falcão, Jéssica Pedroso, Juliana F Mello e Silva, Laura C Rodrigues, Ligia Kerr, Marcia F Almeida, Maria YT Ichihara, Mariana S Felisbino-Mendes, Maurício L Barreto, Naiá Ortelan, Miriam H Tsunemi, Natanael J Silva, Nathalia S Guimarães, Rafaela S Andrade, Rita CO Carvalho-Sauer, Rita C Ribeiro-Silva, Rosemeire L Fiaccone, Ruth Dundas, Sara A Silva, Srinivasa V Katikireddi, Thais RB Carrilho, Walmir M Caminhas.

Word count - abstract: 299/300

Word count - main text: 3592

Number of references: 70

Number of figures: 1

Number of tables: 3

For peer review only - http://bmjopen.bmj.com/site/about/guidelines.xhtml

ABSTRACT

Introduction: There is a limited understanding of the early nutrition and pregnancy determinants of short- and long-term maternal and child health in ethnically diverse and socioeconomically vulnerable populations within low- and middle-income countries. This investigation program aims to: 1. describe maternal weight trajectories throughout the life course; 2. describe child weight, height, and body mass index (BMI) trajectories; 3. create and validate models to predict childhood obesity at five years of age; 4. estimate the effects of pre-pregnancy BMI, gestational weight gain (GWG), and maternal weight trajectories on adverse maternal and neonatal outcomes and child growth trajectories; 5. estimate the effects of pre-pregnancy BMI, GWG, maternal weight, and interpregnancy BMI changes on maternal and child outcomes in the subsequent pregnancy; and 6. estimate the effects of maternal food consumption and infant feeding practices on child nutritional status and growth trajectories. **Methods and analysis:** Linked data from four different Brazilian databases will be used: the 100 million Brazilian Cohort, the Live Births Information System, the Mortality Information System, and the Food and Nutrition Surveillance System. To analyze trajectories, latent-growth, superimposition by translation and rotation (SITAR), and broken stick models will be used. To create prediction models for childhood obesity, machine learning techniques will be applied. For the association between the selected exposure and outcomes variables, generalized linear models will be considered. Directed acyclic graphs will be constructed to identify potential confounders for each analysis investigating potential causal relationships. **Ethics and Dissemination:** This protocol was approved by the Research Ethics Committees of the authors' institutions. The linkage will be carried out in a secure environment. After the linkage, the data will be de-identified, and pre-authorized researchers will access the dataset via a virtual private network connection. Results will

For peer review only

For peer review only - http://bmjopen.bmj.com/site/about/guidelines.xhtml

be reported in open-access journals and disseminated to policymakers and the broader public.

Keywords: Maternal nutrition, gestational weight gain, infant nutrition, growth charts, childhood obesity, nutritional status, food consumption, routinely collected health data.

ARTICLE SUMMARY

Strengths and limitations of this study

- The large sample size and diversity of populations to be used in this study, including data of minorities such as indigenous and *quilombolas* (descendants of people who were slaved), the possibility to stratify the analyses according to several intersecting socioeconomic characteristics, and the use of longitudinal data are unique aspects of this nutrition research program.
- The analytical approaches include cutting-edge methods to analyze growth trajectories, such as the SITAR and the broken stick models, and to create predictive models using machine learning techniques.
- Data from administrative datasets will be analyzed, which were not produced for research and, therefore, may be more prone to measurement errors when compared to data primarily collected for research purposes.
- The study is potentially vulnerable to linkage bias since data from several different administrative systems will be linked.
- The coverage of some national information systems is restricted to socioeconomically disadvantaged populations and varies in quality and completeness across regions of Brazil, which will enable estimates for specific groups, but results may not be generalizable.

For peer review only - http://bmjopen.bmj.com/site/about/guidelines.xhtml

INTRODUCTION

Maternal nutritional status before, during, and after pregnancy has been associated with the risk of adverse outcomes for the infant and the mother (1-4). Pre-pregnancy overweight, excessive gestational weight gain (GWG), and interpregnancy weight increase are associated with outcomes such as the birth of large for gestational age (LGA) infants, neonatal mortality, maternal gestational diabetes, hypertensive disorders, and mortality, and in the long-term, childhood and maternal obesity (1-13). Thus, the increase in the prevalence of such predictors among women of reproductive age in low- and middle-income countries (LMICs) may elevate the risk on the referred outcomes (14). In Brazil, more than 60% of adult women are overweight (15). Thus, the association between maternal nutritional status and adverse outcomes, especially in the medium and long-term, needs to be further explored.

The association of maternal interpregnancy weight change with outcomes in subsequent pregnancies can also be of great importance. However, few studies have explored this relationship due to the need for long-term follow-up (16). Studies show that interpregnancy increases in body mass index (BMI) may increase the risk of short-term outcomes, such as gestational diabetes, hypertensive disorders, cesarean delivery, and thromboembolism in the subsequent pregnancy (9, 17). It has also been shown that BMI reduction can increase the risk of small for gestational age (SGA) birth (17). However, little is known regarding the association of pre-pregnancy BMI, GWG, postpartum weight retention, BMI change during pregnancies, and important long-term adverse outcomes, such as maternal and child mortality and childhood obesity, in developing and socioeconomically vulnerable populations. In addition, maternal weight and child growth patterns and trajectories remain underexplored in these populations, mainly due to the

For peer review only - http://bmjopen.bmj.com/site/about/guidelines.xhtml

lack of longitudinal nationally representative data. It is crucial to identify those trajectories, their determinants, and how they are related.

Furthermore, studies investigating the influence of maternal and infant diet on the nutritional status of children under five based on national population data are also scarce and could help shape maternal and child health services and policy interventions. In general, most available studies evaluated the association between maternal and child diet. Bjerregaard et al. (18) studied 19,582 mother-offspring pairs in the Danish National Birth Cohort. They observed a significant association between the maternal diet quality during pregnancy and the quality of the offspring's diet at the age of 14. The results from another cohort, conducted among 3,422 mothers and children from Portugal, revealed that children whose mothers had worse dietary scores were more likely to follow unhealthier patterns at the age of four (19). Thus, maternal and infant diet seem to be related and to affect child's nutritional statis. However, further investigation to tease apart the complex pathways between the mother and infant's diet and child nutritional status is required.

The use of data from national administrative systems has increased throughout the years (20). The utilization of those data for research purposes has recently received attention with improving methods to analyze big data, enhanced secure storage infrastructure, and increased computational capacity (21, 22). Using data collected as part of the routine of healthcare services can be helpful to monitor trends in health indicators and inform public health policies (23). These data are critical in LMICs, where investment in research is generally scarce. Since 1975, several information systems have been created in Brazil. However, little has been done to harness their resources, especially in the maternal and child nutrition field. Big administrative databases can make it possible to study several maternal and child nutritional health problems which have only been investigated in studies with small sample sizes or not explored at all.

For peer review only - http://bmjopen.bmj.com/site/about/guidelines.xhtml

Maternal nutritional status, diet, and lifestyle, and the family's sociodemographic conditions are all potential determinants of the occurrence of maternal and infant adverse outcomes. However, these relationships were not adequately tested in LMICs. In addition, these are complicated pathways, and causal methods, applied to longitudinal data and through a life course lens, along with large sample sizes, can help in the understanding of those. Thus, the current protocol represents an unprecedented study in a LMIC population, and it differs from currently available studies carried out in small cohorts and with restricted geographical representation. To the best of our knowledge, this is the first initiative to use a population-based linkage database of administrative and health registry data to study maternal and child health and nutritional outcomes in Brazil.

Our objectives in this nutrition research program are to: 1. describe maternal body weight trajectories during the life course (adolescence, adulthood, pregnancy, and postpartum); 2. describe child weight, height, and BMI trajectories until the age of five; 3. create and validate models to predict childhood obesity at the age of five years; 4. Estimate the effects of pre-pregnancy BMI, GWG, maternal weight, and BMI changes between pregnancies, and maternal weight trajectories on adverse maternal and neonatal outcomes and child growth trajectories; 5. estimate the effects of pre-pregnancy BMI, GWG, and maternal weight and BMI changes on adverse maternal and neonatal outcomes in the subsequent pregnancy; 6. estimate the effects of maternal and infant feeding practices (food intake markers during pregnancy, breastfeeding, and complementary feeding) on child nutritional status and growth trajectories. We expect that the results obtained from these studies can be translated into actions to improve women's and children's health care and public health policy, and in the long term, in an improvement in the health indicators in these groups.

For peer review only - http://bmjopen.bmj.com/site/about/guidelines.xhtml

METHODS AND ANALYSIS

Conceptual framework

Our conceptual framework considers maternal nutritional status, diet, and lifestyle, and the family's sociodemographic conditions as potential determinants of the occurrence of adverse maternal and child outcomes (**Figure 1**). The complex relationships underlying this framework have been poorly investigated in LMICs. Most evidence regarding these associations comes from high-income countries or was conducted with small sample sizes in LMIC populations (3, 11, 24).

Data source and linkage process

This protocol was developed following the Reporting of Studies Conducted Using Observational Routinely Collected Health Data (RECORD) guidelines (25). This study will use linked data from four different Brazilian databases: the 100 Million Brazilian Cohort baseline, the Live Births Information System (SINASC, from the Portuguese acronym), the Mortality Information System (SIM), and the Food and Nutrition Surveillance System (SISVAN) (**Table 1**). The 100 Million Brazilian Cohort is a dynamic and open cohort created by the *Centro de Integração de Dados e Conhecimentos para Saúde* (CIDACS) (26). The cohort was designed to study the effect of social determinants and social policies on the health of Brazilian people, mainly those who are beneficiaries of social protection programs. It includes individual data from different national databases from January 2008 to December 2018. The cohort is based on the *Cadastro Único* (CadUnico), a shared registry for more than 20 Brazilian social programs, containing data from families with a monthly income ≤ of three minimum wages (~750 USD). The CadUnico database originates from an extensive questionnaire completed at the time of

For peer review only - http://bmjopen.bmj.com/site/about/guidelines.xhtml

application to federal social programs and contains detailed demographic, economic, and social data at the household and individual levels. The CadUnico database was used to generate the baseline of the 100 Million Brazilian Cohort. More details of this process can be found elsewhere (27, 28). The baseline registers of the cohort comprise 131,697,800 individuals.

The SINASC is the primary national system recording the data available in the "declaration of live birth", legally required for every live birth in Brazil. The declaration is filled in immediately after birth by the health worker who assisted the childbirth. In cases where the delivery was not assisted or performed with a registered professional, the declaration is filled in by the Civil Registry Office with the data provided by the declarant. The declaration form includes data on pregnancy and delivery. Details of the system can be found elsewhere (29). CIDACS has access to identified data of the SINASC between 2001 and 2018, comprising more than 52 million records.

The third database, the SIM, records data from the "declaration of death", which is a document that must be completed by a physician who attests to the cause of death. The form contains information on the identity of the deceased, their place of residency and death, and the cause of death. For infant mortality under 1 year of age, it also provides information on the deceased's mother. The details of the death certificates can be found elsewhere (30). The database available at the CIDACS includes 20,366,604 records between 2001 and 2018.

Finally, the fourth database is the SISVAN, which monitors the Brazilian population's nutritional status since 2008 (31). The system was designed to record individual sociodemographic, anthropometric, and food intake data of subjects using public healthcare services in all life-course stages. The main goal of the SISVAN is to inform the evaluation and development of public health nutrition policies. The dataset

For peer review only - http://bmjopen.bmj.com/site/about/guidelines.xhtml

available at CIDACS includes 307,245,508 records (59,724,164 individuals) in all the life cycles from 2008 – 2017. Women of all ages and children aged up to 10 years correspond to 76.4% (235,801,691) and 37.6% (115,510,621) of the records in the SISVAN.

For the work described in this protocol, we will link the 100 Million Cohort baseline data with the data from the SISVAN, SIM, and SINASC. The 100 Million Brazilian Cohort will form the population spine for linkage. Two approaches will be used to perform record linkage between the four sources: **a.** deterministic linkage; **b.** non-deterministic linkage based on the similarity index. Deterministic linkage will be performed between the 100 Million Brazilian Cohort baseline and the SISVAN because both datasets contain the Social Information Number (NIS), a unique identifier assigned for each individual. Linkage of the other administrative datasets will occur using a non-deterministic approach. This method will also link the data from the cohort with the subset of individuals in the SISVAN with no NIS. To do that, CIDACS created a tool (CIDACS-RL) to link individual records based on identifiers, such as name, sex, age or date of birth, mother's name, and the municipality of residence (32, 33). The CIDACS-RL applies indexing and searching algorithms implemented in the Apache Lucene solution as the blocking strategy. The indexation strategy allows the CIDACS-RL to search for the most similar records from the indexed dataset for each record in the main dataset and submit them to the pairwise comparisons step. Candidate linking records are ordered by the similarity scores, and only the comparison pair with the highest score is retained as a potential link. All remaining candidate records are discarded (32, 34). The CIDACS-RL tool was previously validated and presented higher accuracy when compared to other linkage tools (33).

The final linked dataset will be submitted to an extensive data structuring process before the analyses are performed. We will first identify the mother-child pairs in the 100

For peer review only - http://bmjopen.bmj.com/site/about/guidelines.xhtml

Million Brazilian Cohort baseline based on the child's family ID number and birth information, such as the mother's name and date of birth. This identification enables the structuration of the dataset and the link between the mother and child data. This step is essential because the mother-child pairs are not automatically identified in the linkage.

Main variables

The main variables for analyses are summarized in **Table 2**. Several maternal and child variables can be created with the linked datasets. These variables will be analyzed as outcomes or exposures/confounders for several objectives proposed in this protocol. It is important to mention that, whenever possible, we plan to explore those variables as continuous and categorical, according to the proposed cutoffs. For example, children's BMI will be tested as a continuous score and categorized into underweight, normal, overweight, and obesity. Other essential variables to achieve the proposed objectives include socioeconomic and demographic conditions, newborn, mother, pregnancy, and delivery characteristics (**Table 1**).

For peer review only

Analytical approaches

The final linked dataset will be submitted to an extensive data-cleaning process before the analyses are performed. In this step, we plan to implement a routine to identify implausible anthropometric values considering cross-sectional and longitudinal data. The approaches proposed by Yang and Hutcheon (35), based on conditional centiles; Shi et al. (36), based on jackknife residuals; and the method based on linear mixed spline regression proposed by Welch et al. (37) and Boone-Heinonen et al. (38) will be considered. In addition, modifications of the methods to deal with cross-sectional data (39) and methods to identify populational outliers based on *z* scores of reference charts

For peer review only - http://bmjopen.bmj.com/site/about/guidelines.xhtml

will also be considered (38). After the identification, we plan to perform sensitivity analyses to assess the impact of removing the observations flagged as outliers from the dataset.

Finally, because Brazil is a remarkably diverse country, we plan to perform a heterogeneity assessment of the main variables to be used in the studies across the states and regions to ensure we are working with homogeneous data. Two approaches will be considered in this step: a. the use of multilevel models to calculate the proportion of the variance in the variable of interest that could be attributable to the origin of the data (39), in this case, the region of residence; b. the standardized site difference method, as proposed by the WHO in the Multicentre Study (40). This evaluation of heterogeneity will also capture aspects related to the quality and coverage of the dataset, which will be considered before deciding the next steps of the analyses.

We plan to conduct descriptive analyses initially, including the presentation of summary measurements (mean, standard deviations, median, interquartile range, kurtosis, and skewness measurements) for continuous variables and absolute (n) and relative (%) frequencies for categorical ones. A summary of the proposed analytical approaches for each goal is available in **Table 3**.

Because we plan to work with large sample sizes, it will be possible to adopt an intersectionality perspective, exploring how combinations of social characteristics (such as gender and race/ethnicity or socioeconomic position and race/ethnicity) are related to outcomes of interest (41). We will do that by stratifying the analyses according to several variables, such as those identifying different social contexts. For example, we will be able to stratify the analyses by Brazilian municipality, identifying clusters of richer (closer to the European reality) and poorer (closer to Sub-Saharan Africa context) populations, and

For peer review only - http://bmjopen.bmj.com/site/about/guidelines.xhtml

explore how combinations of family size, race/ethnicity, urban/rural context influence the outcomes.

To describe trajectories in maternal weight and child weight, height, and BMI, we plan to test three different classes of models: latent class growth, broken stick, and Super Imposition by Translation And Rotation (SITAR) models. The novelty of this study is the possibility of stratifying the trajectories according to several sociodemographic and economic variables, such as race/ethnicity and participation in conditional cash-transfer programs.

We plan to use latent-class growth models to identify trajectory groups that can be later associated with risk factors (42). Those models allow longitudinal data to inform clusters of individuals following different patterns of change over time, and they account for varying distances between the measurements. The number of trajectory groups can be selected based on Bayesian information criteria (42).

The *broken stick* models are another recent approach to be used to model growth trajectories. These models are a particular case of the linear mixed model. They summarize irregular individual trajectories, i.e., non-equidistant measurements over time, by estimates made at a pre-specified time interval (43). The models were initially developed to facilitate the statistical analysis and the testing of critical ages in the onset of childhood obesity, but several other applications are possible (43, 44).

Finally, we plan to test the SITAR models. These models describe each trajectory in three biologically interpretable parameters: size, time, and velocity. By using them, we may estimate each child's variation from the curve expressed by child-specific random effects estimates for absolute amount, timing, and acceleration (45). These three child-specific estimates can be used as exposure variables to explore associations with adverse outcomes (46). It is important to mention that those three techniques are used for different

For peer review only - http://bmjopen.bmj.com/site/about/guidelines.xhtml

goals, which should be considered when choosing the final model. To select the final model to create the trajectories that will be used in the subsequent studies, we will consider the group's existing knowledge and experts' opinions.

To construct predictive models for childhood obesity at the age of five, to identify factors that could be used to target interventions, we plan to consider artificial intelligence models, such as those based on Artificial Neural Networks and Adaptive Fuzzy Systems (47, 48). The risk and protective factors to be considered in these models are the characteristics of the child, characteristics of the mother, and family/environment factors. The following steps will be executed to create and validate the models: a. data aggregation and pre-processing (filtering and eliminating inconsistencies); b. data separation in sets of training, validation, and testing; c. training of the models; and d. results, validation, and testing of the models. The performance of the models will be evaluated through the measures of sensitivity, specificity, positive and negative predictive values, overall accuracy, and the area under the Receiver Operating Characteristic (ROC) curves. Experts will also validate the results based on documents and reference frameworks for child obesity assessment. We will also assess the performance of the algorithm for different population subgroups, particularly from an equity perspective, to identify how it works among more socioeconomically disadvantaged and minority ethnic groups.

To estimate the effects of pre-pregnancy BMI, GWG, maternal weight, and BMI changes between pregnancies, and maternal weight trajectories on the selected outcomes, we plan to use generalized linear models (GLMs). For categorical outcomes, logistic and Poisson regressions with robust variance will be considered. For continuous outcomes, linear regression will be used. We also plan to explore quantile regression for continuous outcomes. If the exposure is a longitudinal variable, we plan to account for the intra-subject variability by running linear mixed-effects models to extract the best linear

For peer review only

unbiased prediction (BLUP). This longitudinal prediction of the exposures can then be incorporated into a GLM model (49). Whenever the outcome is longitudinal, linear mixed-effects models will be used. We will consider the distribution of the variables and perform transformations whenever necessary. In some situations, we may also consider modeling the data with restricted cubic splines (50) or fractional polynomials (51, 52) to account for the non-linearity of the relationships between the given variables. After adjusting the models, we plan to run the proper diagnostics to evaluate the goodness of fit.

To define the set of confounders to be used in the analyses investigating potential causal relationships, we plan to construct directed acyclic graphs (DAGs) using the program dagitty (http://www.dagitty.net/). The use of DAGs is becoming quite common in the nutrition field due to their ability to portray the assumptions about the relationships between the variables in the causal structures and establishing the minimal set of variables sufficient for adjusting that blocks all backdoor paths and does not open any closed pathways (53). The DAGs are particularly useful to identify the minimal set of confounders for adjustment in the regression models. In the cases where we plan to have the analyses stratified (according to socioeconomic conditions, for example), a DAG will be constructed for each subgroup. The consideration of potential confounders will be informed by the existing literature and substantive knowledge of the investigators. We also plan to apply sensitivity analyses to consider the impact of unmeasured confounding (54).

In the studies aiming to test associations between maternal and child characteristics, it is crucial to deal with missing data in the variables included in the models as exposure and confounders. Hence, we plan to consider the mechanism underlying the occurrence of missing data (whether missingness occurred completely at

For peer review only - http://bmjopen.bmj.com/site/about/guidelines.xhtml

For peer review only

random – MCAR, at random – MAR, or not at random - MNAR), the pattern of missingness (monotonic or non-monotonic), and the most appropriate method to deal with each of those. Techniques such as multiple imputation, inverse probability weighting, and full-information maximum likelihood will be considered (55, 56). We also plan to follow the recently published 'Framework for the treatment and reporting of missing data in observational studies' (TARMOS framework) to deal with missing data in a study-based approach (57). All the proposed analyses will be performed in R, Stata, or Python. The latter is important for the development of the aforementioned artificial intelligence models.

The work described in this protocol was initiated in 2023. The data linkage of several of the databases was already conducted, with a success rate of over 75% in the linkage, but others are still to be done in the following months. The complete timeline for the development of this protocol is five years.

Patient and public involvement

Patients and the public were not involved in any step of this study.

ETHICS AND DISSEMINATION

The objectives described in this protocol were approved by the Research Ethics Committees from the Gonçalo Moniz Institute/Oswaldo Cruz Foundation (CAAE: 56003716.0.0000.0040), the Institute of Collective Health/Federal University of Bahia (CAAE: 41695415.0.0000.5030), the Federal University of Rio de Janeiro (CAAE: 85914318.2.0000.5275; 18447919.3.0000.5264), and the Federal University of Minas

For peer review only - http://bmjopen.bmj.com/site/about/guidelines.xhtml

Gerais (CAAE: 37534620.3.0000.5149). To ensure data privacy and confidentiality, all data linkage procedures will be conducted in a physically and virtually secure environment and follow strict internal information security measures (26). Access to linked and de-identified datasets will be granted to authorized researchers only. The analysis will be performed in the CIDACS data analysis environment, a safe virtual infrastructure that provides remote data access and analysis tools. The final linked and de-identified datasets will receive a Digital Object Identifier (DOI), and the complete specification of how the dataset was created will be made available upon request. Manuscripts from studies of this protocol will be submitted to open-access journals. In addition, we will promote stakeholder events and produce policy briefs to disseminate the results to policymakers and the broader public.

AUTHOR CONTRIBUTION

TRBC and NJS contributed equally to this paper and share the first authorship. GK, RCRS and MLB share the senior authorship. TRBC, NJS, and GK wrote the manuscript with input from ESP, IRF, RFL, LCR, SVK, AHL, RD, AP, GVM, RCRS, and MLB. GK, GVM, and RCRS are the main responsible for the design of the research. RCRS is the coordinator of the GeNut-CIDACS group. MLB is the coordinator of the CIDACS and the responsible to grant access to the datasets. The authors from the GeNut-CIDACS group contributed to the revision of the manuscript. TRBC, NJS, and GK had primary responsibility for the final content. All authors read and approved the final manuscript.

FUNDING STATEMENT

For peer review only

For peer review only - http://bmjopen.bmj.com/site/about/guidelines.xhtml

This study is funded by the Brazilian Ministry of Health (TED 64/2022, process 25000.148278/2022-10). CIDACS receives core support from the Wellcome Trust (202912/Z/16/Z), the Brazilian Health Surveillance Secretariat, the Brazilian Ministry of Health, the State of Bahia, the Research Support Foundation of the State of Bahia (FAPESB), the Research and Project Funding Agency (FINEP), and the Secretariat of Science and Technology of the State of Bahia (SECTI). SVK acknowledges funding from a NRS Senior Clinical Fellowship (SCAF/15/02), the Medical Research Council (MC_UU_00022/2), and the Scottish Government Chief Scientist Office (SPHSU17). NJS acknowledges support from the Spanish Ministry of Science and Innovation and State Research Agency through the "Centro de Excelencia Severo Ochoa 2019-2023" Program (CEX2018-000806-S) and support from the Generalitat de Catalunya through the CERCA Program.

COMPETING INTERESTS

The authors declare that they have no competing interests.

For peer review only

For peer review only - http://bmjopen.bmj.com/site/about/guidelines.xhtml

REFERENCES

1. Aune D, Saugstad OD, Henriksen T, Tonstad S. Maternal body mass index and the risk of fetal death, stillbirth, and infant death: a systematic review and meta-analysis. JAMA 2014;311(15):1536-46.

2. Adane AA, Shepherd CCJ, Lim FJ, et al. The impact of pre-pregnancy body mass index and gestational weight gain on placental abruption risk: a systematic review and meta-analysis. Arch Gynecol Obstet 2019;300(5):1201-10.

3. Goldstein RF, Abell SK, Ranasinha S, et al. Association of gestational weight gain with maternal and infant outcomes: a systematic review and meta-analysis. JAMA 2017;317(21):2207-25.

4. Saucedo M, Esteves-Pereira AP, Pencolé L, et al. Understanding maternal mortality in women with obesity and the role of care they receive: a national case-control study. Int J Obes 2021;45(1):258-65.

5. Gaillard R, Steegers EA, Franco OH, et al. Maternal weight gain in different periods of pregnancy and childhood cardio-metabolic outcomes. The Generation R Study. Int J Obes 2015;39(4):677-85.

6. Gaillard R, Welten M, Oddy WH, et al. Associations of maternal prepregnancy body mass index and gestational weight gain with cardio-metabolic risk factors in adolescent offspring: a prospective cohort study. BJOG 2016;123(2):207-16.

7. Voerman E, Santos S, Inskip H, et al. Association of gestational weight gain with adverse maternal and infant outcomes. JAMA 2019;321(17):1702-15.

8. Voerman E, Santos S, Patro Golab B, et al. Maternal body mass index, gestational weight gain, and the risk of overweight and obesity across childhood: An individual participant data meta-analysis. PLoS Med 2019;16(2).

For peer review only - http://bmjopen.bmj.com/site/about/guidelines.xhtml

9. Ehrlich SF, Hedderson MM, Feng J, et al. Change in body mass index between pregnancies and the risk of gestational diabetes in a second pregnancy. Obstet Gynecol 2011;117(6):1323-30.

10. Brunner S, Stecher L, Ziebarth S, et al. Excessive gestational weight gain prior to glucose screening and the risk of gestational diabetes: a meta-analysis. Diabetologia 2015;58(10):2229-37.

11. Oteng-Ntim E, Mononen S, Sawicki O, Seed P, Bick D, Poston L. Interpregnancy weight change and adverse pregnancy outcomes: A systematic review and meta-analysis. BMJ Open 2018;8:e018778.

12. Rogozinska E, Zamora J, Marlin N, et al. Gestational weight gain outside the Institute of Medicine recommendations and adverse pregnancy outcomes: analysis using individual participant data from randomised trials. BMC Pregnancy Childbirth 2019;19(1):322.

13. Teulings NEWD, Masconi KL, Ozanne SE, et al. Effect of interpregnancy weight change on perinatal outcomes: systematic review and meta-analysis. BMC Pregnancy Childbirth 2019;19(1):386.

14. Poston L, Caleyachetty R, Cnattingius S, et al. Preconceptional and maternal obesity: epidemiology and health consequences. Lancet Diabetes Endocrinol 2016;4(12):1025-36.

15. IBGE. Instituto Brasileiro de Geografia e Estatística. Pesquisa nacional de saúde 2019: atenção primária à saúde e informações antropométricas. Rio de Janeiro: IBGE; 2020.

16. Taylor EJ, Wilding S, Ziauddeen N, et al. Change in modifiable maternal characteristics and behaviours between consecutive pregnancies and offspring adiposity: A systematic review. Obes Rev 2020;21(11):e13048.

For peer review only

For peer review only - http://bmjopen.bmj.com/site/about/guidelines.xhtml

17. Wallace JM, Bhattacharya S, Horgan GW. Weight change across the start of three consecutive pregnancies and the risk of maternal morbidity and SGA birth at the second and third pregnancy. PLoS One 2017;12(6):e0179589.

18. Bjerregaard AA, Halldorsson TI, Tetens I, Olsen SF. Mother's dietary quality during pregnancy and offspring's dietary quality in adolescence: Follow-up from a national birth cohort study of 19,582 mother-offspring pairs. PLoS Med 2019;16(9):e1002911.

19. Durão C, Severo M, Oliveira A, et al. Association of maternal characteristics and behaviours with 4-year-old children's dietary patterns. Matern Child Nutr 2017;13(2).

20. Daymont C, Ross ME, Russell Localio A, et al. Automated identification of implausible values in growth data from pediatric electronic health records. J Am Med Inform Assoc 2017;24(6):1080-7.

21. Murdoch TB, Detsky AS. The inevitable application of big data to health care. JAMA 2013;309(13):1351-2.

22. Mooney SJ, Westreich DJ, El-Sayed AM. Commentary: Epidemiology in the era of big data. Epidemiology. 2015;26(3):390-4.

23. Harron K, Dibben C, Boyd J, et al. Challenges in administrative data linkage for research. Big Data Soc 2017;4(2):2053951717745678.

24. Victora CG, Barros FC. Cohorts in low- and middle-income countries: from still photographs to full-length movies. J Adolesc Health 2012;51(6 Suppl):S3-4.

25. Benchimol EI, Smeeth L, Guttmann A, et al. The REporting of studies Conducted using Observational Routinely-collected health Data (RECORD) statement. PLoS Med 2015;12(10):e1001885.

26. Barreto ML, Rodrigues L, Denaxas S, et al. The Center for Data and Knowledge Integration for Health (CIDACS): an experience of linking health and social data in Brazil. Int J Popul Data Sci 2019;4(2).

For peer review only - http://bmjopen.bmj.com/site/about/guidelines.xhtml

27. Pescarini JM, Craig P, Allik M, et al. Evaluating the impact of the Bolsa Familia conditional cash transfer program on premature cardiovascular and all-cause mortality using the 100 million Brazilian cohort: a natural experiment study protocol. BMJ Open 2020;10(11):e039658.

28. Paixao ES, Cardim LL, Falcao IR, et al. Cohort Profile: Centro de Integracao de Dados e Conhecimentos para Saude (CIDACS) Birth Cohort. Int J Epidemiol 2021;50(1):37-8.

29. Szwarcwald CL, Leal MDC, Esteves-Pereira AP, et al. [Evaluation of data from the Brazilian Information System on Live Births (SINASC)]. Cad Saude Publica 2019;35(10):e00214918.

30. Jorge MHPdM, Laurenti R, Gotlieb SLD. Análise da qualidade das estatísticas vitais brasileiras: a experiência de implantação do SIM e do SINASC. Cien Saude Colet 2007;12:643-54.

31. Brasil. Ministério da Saúde. Secretaria de Atenção à Saúde. Departamento de Atenção Básica. Política Nacional de Alimentação e Nutrição. In: Ministério da Saúde. Secretaria de Atenção à Saúde. Departamento de Atenção Básica. Brasília: Ministério da Saúde; 2013. p. 84.

32. Ali MS, Ichihara MY, Lopes LC, et al. Administrative Data Linkage in Brazil: Potentials for Health Technology Assessment. Front Pharmacol 2019;10:984.

33. Barbosa GCG, Ali MS, Araujo B, et al. CIDACS-RL: a novel indexing search and scoring-based record linkage system for huge datasets with high accuracy and scalability. BMC Med Inform Decis Mak 2020;20(1):289.

34. Almeida D, Gorender D, Ichihara MY, et al. Examining the quality of record linkage process using nationwide Brazilian administrative databases to build a large birth cohort. BMC Med Inform Decis Mak 2020;20(1):173.

For peer review only

For peer review only - http://bmjopen.bmj.com/site/about/guidelines.xhtml

35. Yang S, Hutcheon JA. Identifying outliers and implausible values in growth trajectory data. Ann Epidemiol 2016;26(1):77-80 e1-2.

36. Shi J, Korsiak J, Roth DE. New approach for the identification of implausible values and outliers in longitudinal childhood anthropometric data. Ann Epidemiol 2018;28(3):204-11 e3.

37. Welch C, Petersen I, Walters K, et al. Two-stage method to remove population- and individual-level outliers from longitudinal data in a primary care database. Pharmacoepidemiol Drug Saf 2012;21(7):725-32.

38. Boone-Heinonen J, Tillotson CJ, O'Malley JP, et al. Not so implausible: impact of longitudinal assessment of implausible anthropometric measures on obesity prevalence and weight change in children and adolescents. Ann Epidemiol 2019;31:69-74 e5.

39. Carrilho TRB, Farias DR, Batalha MA, et al. Brazilian Maternal and Child Nutrition Consortium: establishment, data harmonization and basic characteristics. Sci Rep 2020;10(1):14869.

40. WHO Multicentre Growth Reference Study Group. Assessment of differences in linear growth among populations in the WHO Multicentre Growth Reference Study. Acta Paediatr Suppl 2006;95(S450):56-65.

41. Jackson JW, VanderWeele TJ. Intersectional decomposition analysis with differential exposure, effects, and construct. Soc Sci Med 2019;226:254-9.

42. Leonard SA, Rasmussen KM, King JC, Abrams B. Trajectories of maternal weight from before pregnancy through postpartum and associations with childhood obesity. Am J Clin Nutr 2017;106(5):1295-301.

43. Buuren Sv. Broken stick model for irregular longitudinal data. J Stat Softw. 2021.

44. Anderson C, Hafen R, Sofrygin O, et al. Comparing predictive abilities of longitudinal child growth models. Stat Med 2019;38(19):3555-70.

For peer review only

45. Cole TJ, Donaldson MD, Ben-Shlomo Y. SITAR--a useful instrument for growth curve analysis. Int J Epidemiol 2010;39(6):1558-66.

46. Riddell CA, Platt RW, Bodnar LM, Hutcheon JA. Classifying gestational weight gain trajectories using the SITAR growth model. Paediatr Perinat Epidemiol 2017;31(2):116-25.

47. Rumelhart DE, Hinton GE, Williams RJ. Learning representations by back-propagating errors. Nature 1986;323(6088):533-6.

48. Jang JR. ANFIS: adaptive-network-based fuzzy inference system. IEEE Trans Syst Man Cybern 1993;23(3):665-85.

49. Chen YH, Ferguson KK, Meeker JD, et al. Statistical methods for modeling repeated measures of maternal environmental exposure biomarkers during pregnancy in association with preterm birth. Environ Health 2015;14:9.

50. Harrell FE, Jr., Lee KL, Pollock BG. Regression models in clinical studies: determining relationships between predictors and response. J Natl Cancer Inst 1988;80(15):1198-202.

51. Sauerbrei W, Royston P. Building multivariable prognostic and diagnostic models: transformation of the predictors by using fractional polynomials. J R Stat Soc Series A 1999;162(1):71-94.

52. Royston P, Ambler G, Sauerbrei W. The use of fractional polynomials to model continuous risk variables in epidemiology. Int J Epidemiol 1999;28(5):964-74.

53. Greenland S, Pearl J, Robins JM. Causal diagrams for epidemiologic research. Epidemiology 1999;10(1):37-48.

54. VanderWeele TJ, Ding P. Sensitivity analysis in observational research: Introducing the E-Value. Ann Intern Med 2017;167(4):268-74.

For peer review only

For peer review only - http://bmjopen.bmj.com/site/about/guidelines.xhtml

55. Little TD, Jorgensen TD, Lang KM, Moore EW. On the joys of missing data. J Pediatr Psychol 2014;39(2):151-62.

56. Huque MH, Carlin JB, Simpson JA, Lee KJ. A comparison of multiple imputation methods for missing data in longitudinal studies. BMC Med Res Methodol 2018;18(1):168.

57. Lee KJ, Tilling KM, Cornish RP, et al. Framework for the treatment and reporting of missing data in observational studies: The Treatment And Reporting of Missing data in Observational Studies framework. J Clin Epidemiol 2021;134:79-88.

58. Lima EE, Queiroz BL. Evolution of the deaths registry system in Brazil: associations with changes in the mortality profile, under-registration of death counts, and ill-defined causes of death. Cad Saude Publica 2014;30(8):1721-30.

59. Carrilho TRB, Rasmussen KM, Farias DR, et al. Agreement between self-reported pre-pregnancy weight and measured first-trimester weight in Brazilian women. BMC Pregnancy Childbirth. 2020;20(1):734.

60. WHO Expert Committee on Physical Status. Physical status: the use and interpretation of anthropometry: report of a WHO Expert Committee. Geneva: World Health Organization; 1995. x, 452 p. p.

61. Pole JD, Dodds LA. Maternal outcomes associated with weight change between pregnancies. Can J Public Health 1999;90(4):233-6.

62. Oteng-Ntim E, Mononen S, Sawicki O, et al. Interpregnancy weight change and adverse pregnancy outcomes: a systematic review and meta-analysis. BMJ Open 2018;8(6):e018778.

63. Martinez-Hortelano JA, Berlanga-Macias C, Pozuelo-Carrascosa DP, et al. Interpregnancy weight change and perinatal outcomes: A protocol for a systematic review and meta-analysis. Medicine 2019;98(20):e15470.

For peer review only - http://bmjopen.bmj.com/site/about/guidelines.xhtml

For peer review only

64. Amorim AR, Linne Y, Kac G, Lourenco PM. Assessment of weight changes during and after pregnancy: practical approaches. Matern Child Nutr 2008;4(1):1-13.

65. Kac G, Carrilho TRB, Rasmussen KM, et al. Gestational weight gain charts: results from the Brazilian Maternal and Child Nutrition Consortium. Am J Clin Nutr 2021;113(5):1351-60.

66. World Health Organization. ICD-10: International Statistical Classification of Diseases and Related Health Problems, 10th Revision. Geneva: WHO; 2010.

67. Villar J, Cheikh Ismail L, Victora CG, et al. International standards for newborn weight, length, and head circumference by gestational age and sex: the Newborn Cross-Sectional Study of the INTERGROWTH-21st Project. Lancet 2014;384(9946):857-68.

68. World Health Organization. WHO child growth standards: length/height-for-age, weight-for-age, weight-for-length, weight-for-height and body mass index-for-age: methods and development. Geneva: World Health Organization; 2006.

69. Brasil. Ministério da Saúde. Orientações para a coleta e análise de dados antropométricos em serviços de saúde. Norma Técnica do Sistema de Vigilância Alimentar e Nutricional - SISVAN. Brasília: Ministério da Saúde; 2011.

70. Monteiro PO, Victora CG. Rapid growth in infancy and childhood and obesity in later life - a systematic review. Obes Rev 2005;6(2):143-54.

For peer review only - http://bmjopen.bmj.com/site/about/guidelines.xhtml

TABLES

Table 1. Variables available in each data source to be used in the maternal and child nutrition research program.

Database	Data collection process	Available variables	Coverage of the database	Period available
100 Million Brazilian Cohort baseline	The baseline of the 100 million Brazilian cohort was created using administrative records from individuals ≥ 16 years of age whose families applied for social assistance via the *Cadastro Único para Programas Sociais*.	Characteristics of the registered individuals (year of application/entry, gender, age at entry year, self-declared race/ethnicity, education, marital status, head of the family), characteristics of the household (municipality of residence, urban/rural area of residence, household building material, water supply, sewage disposal, waste disposal/garbage collection, electricity, number of individuals in the household, number of rooms in the household); 'Bolsa Familia*' information (identification of beneficiary families or not, receipt start date, final receipt date, receipt time in days).	More than 50% of the Brazilian population	2001 - 2018
SINASC (Live Births Information System)	The form (declaration of live birth) is completed by a health professional present at the delivery, and the system records information about all live births in Brazil.	Characteristics of the newborn (sex; Apgar score in the 1st and 5th minute, birth weight, presence of abnormalities, congenital anomalies identified at birth using the ICD-10 code), place of birth, characteristics of the mother (name, age, marital status, education, race, place of residence), father's age, characteristics of pregnancy and delivery (number of previous pregnancies of live births, stillbirth or abortion, length of gestation, type of delivery, number of fetuses, number of visits to prenatal care facilities, month of pregnancy in which prenatal care started, which health professionals were present at the delivery).	Approximately 97% of the Brazilian live births	2001 – 2018

For peer review only - http://bmjopen.bmj.com/site/about/guidelines.xhtml

SIM (Mortality Information System)	The form (declaration of death) is completed by the physician who attests the cause of death. The system records all deaths in Brazil, and it differentiates between fetal and non-fetal deaths.	Characteristics of the dead person (date of death and birth, name, name of the mother and father, sex, race, marital status, occupation and education, address), identification of the place of death (hospital, home, public place and includes the address of the place), characteristics of the mother (name, age, marital status, education, occupation, race, number of births, place of residence, length of gestation, number of previous stillbirths or abortions, type of delivery, number of fetus in the current pregnancy – filled in case of fetal death or infant mortality), birth weight (filled only in case of fetal deaths or infant mortality), and cause of death using ICD-10 code.	SIM coverage has been improving over time, reaching around 95% in some regions**.	2011 – 2018
SISVAN (Food and Nutrition Surveillance System)	The forms are completed by primary care workers in the public healthcare system. Anthropometric and food intake markers data of the Brazilian Unified Health System (SUS) users are collected.	Date of birth, age; sex, race/ethnicity, education, participation in social/health programs, diseases, and intercurrences, food intake data (food markers according to each stage of the lifecycle), and anthropometric data: weight and height, birth weight for children under two years, waist circumference for adults, calf circumference for elderly, and pre-pregnancy weight for pregnant women;	About 30% among under-five children and 17% among pregnant women	2008 - 2017

*Bolsa Familia is the conditional cash transfer program implemented in Brazil since 2004. ** In 2010, the system's coverage was estimated to be 95% in some Brazilian regions (58).

For peer review only - http://bmjopen.bmj.com/site/about/guidelines.xhtml

Table 2. Main variables to be used in the maternal and child nutrition research program.

Variable	Characteristics
Maternal variables	
Mode of delivery	• It is collected as vaginal or cesarean.<br>• Since 2011, there is also the information if the labor was induced.
Maternal pre-pregnancy BMI	• It is calculated based on weight (kg) and height (m) using the formula: BMI weight (kg)/height $(m)^2$.<br>• Self-reported pre-pregnancy weight will be used for its calculation, considering the good agreement observed between self-reported and measured first-trimester weight in a previous study using the SISVAN data (59).<br>• Continuous variable (kg/m^2).<br>• The WHO cutoffs will be used: underweight (< 18.5 kg/m^2), normal weight (≥ 18.5 and < 25.0 kg/m^2), overweight (≥ 25.0 and < 30.0 kg/m^2) and obesity (≥ 30.0 kg/m^2) (60).
Maternal weight changes between pregnancies	• It is calculated as the difference in self-reported pre-pregnancy weights between consecutive pregnancies.<br>• Continuous variable (kg).<br>• Percentage of weight change (61).
Maternal BMI change between pregnancies	• It is calculated as the difference between BMIs at the beginning of consecutive pregnancies.<br>• It will be used as a continuous variable (units of increase/decrease in BMI) or categorized (62).<br>• The change in BMI category (based on the WHO cutoffs) between pregnancies will also be used (63).
Gestational weight gain (GWG)	• Several ways to calculate GWG will be tested in this study.<br>• Total GWG is calculated as the difference between the weight measured up to 14 days before delivery and self-reported pre-pregnancy weight (64).<br>• Cumulative GWG is calculated as the difference between GWG measured in prenatal visits and self-reported pre-pregnancy weight (64).<br>• Continuous variable (kg).<br>• Z scores of the Brazilian GWG charts (65).<br>• GWG per trimester can also be considered.
Postpartum weight retention	• It is calculated as the difference between the weight measured at 6 and 12 months postpartum and self-reported pre-pregnancy weight.<br>• Continuous (kg) variable.<br>• Categorical variable – we plan to test several cutoffs for its classification.
Maternal weight trajectories	• This outcome will be created in objective 1, and the groups of trajectories identified will be used in the following studies.
Food intake markers	• The following markers are available: consumption of beans; fresh fruits; vegetables; hamburgers or processed, mixed meat products; sugar-sweetened beverages; instant noodles, packaged snacks, or crackers; stuffed biscuits/cookies or candies.<br>• The response options are 'yes/no', and the time frame always refers to the intake on the day before the visit. These markers are collected before, during, and after pregnancy.
Maternal mortality	• Maternal death will be defined as the death of women during pregnancy or up to 42 days after delivery, due to any cause related to or aggravated by the pregnancy, but not due to accidental or incidental causes (66).
Child variables	
Gestational age at birth	• This information is available in weeks since 2011. Before, it is available only categorized as <22 weeks, 22 to 27 weeks; 28 to 31 weeks; 32 to 36 weeks; 37 to 41 weeks; and 42 weeks or more.

For peer review only - http://bmjopen.bmj.com/site/about/guidelines.xhtml

	• It can be used in weeks or classified as preterm (< 37 weeks)/term birth (≥ 37 weeks) (66).<br>• Preterm birth can also be divided into extremely preterm (<28 weeks), very preterm (28–<32 weeks), and moderate or late preterm (32–<37 completed weeks)(66).
Birth weight	• Continuous, in grams<br>• Classified as large (> P90) and small (< P10) for gestational age, according to Intergrowth-21st sex and gestational age-specific charts (67).<br>• Classified as low birth weight (LBW, < 2500g) and macrosomia (>4000g) (68).<br>• LBW can also be classified into very LBW (<1500g) and extremely LBW (< 1000g)(66).
BMI up to 5 years old	• It is calculated based on measured weight (kg) and height (m).<br>• Continuous variable (kg/m²).<br>• BMI-for-age z-scores for sex according to the WHO charts (68) will be used.<br>• It can also be used categorized as proposed by the Brazilian Ministry of Health (69): Underweight (BMI-for-age<-2 z score); Normal (BMI-for-age≥-2 and ≤ 2 z score); Overweight (BMI-for-age>2 and ≤ 3 z score); Obesity (BMI-for-age>3 z score).
Rapid infant growth	• It is determined based on weight-for-age z scores according to the WHO charts (68).<br>• It will be classified when the z scores in weight-for-age between two different ages up to 5 years old is > 0.67 (70).
Growth trajectories	• This outcome will be created in objective 2, and the groups of trajectories identified will be used in the following studies.
Food intake markers	The following markers are collected:<br>• For children < 6 months old: consumption of breast milk; porridge; water/ tea; cow milk; infant formula; fruit juice; fruits; salt food; other food/ beverages.<br>• For children 6 – 23 months old: consumption of breast milk; whole fruit in pieces or mashed (and the daily frequency); salt food (and the daily frequency and how the food was offered - in pieces/ kneaded/ passed through a sieve/ blended/ only the broth); milk other than breast milk; milk porridge; yogurt; vegetables; orange-colored vegetables (carrots, pumpkin, papaya) or fruit or dark green leaves (spinach, kale); leaf greenery (lettuce, cabbage); meat (beef, chicken, pork, fish) or eggs; liver; beans; rice, potatoes, yams, cassava, cassava flour, or pasta; hamburger and/or sausages; sugar-sweetened beverages; instant noodles, packaged snacks, or crackers; stuffed biscuits/cookies or candies.<br>• For children > 24 months old: consumption of beans; fresh fruits; vegetables; hamburgers or processed, mixed meat products; sugar-sweetened beverages; instant noodles, packaged snacks, or crackers; stuffed biscuits/cookies or candies.<br>• The response options are 'yes/no', and the time frame always refers to the intake on the day before the visit.
Child mortality	• Child mortality refers to the death of children under five years old (66).<br>• It is also possible to work with perinatal, neonatal, and postneonatal mortality.<br>• Perinatal mortality includes deaths in the first week of life and fetal deaths (stillbirths) (66).<br>• Neonatal mortality refers to deaths that occurred up to 28 days after birth. It can be divided into two components: a. early neonatal mortality, which includes the deaths among live births in the first 6 days of life; b. late neonatal mortality includes deaths among live births between 7 and 27 days of life (66).<br>• Post neonatal mortality refers to the deaths among live births between 28 and 364 completed days of life (66).

Notes: BMI: Body mass index; GWG: Gestational weight gain; SISVAN: Food and Nutrition Surveillance System; WHO: World Health Organization.

For peer review only - http://bmjopen.bmj.com/site/about/guidelines.xhtml

Table 3. Summary of objectives, outcomes, and proposed analytic approaches.

Objectives	Outcomes/main variables	Analytical approaches
1. Describe maternal body weight trajectories during the life course (adolescence, adulthood, pregnancy, and postpartum).	Maternal weight, age, and gestational age/number of postpartum days in each visit.	• Latent-class growth models<br>• Broken stick models<br>• SITAR models
2. Describe child weight, height, and BMI trajectories until the age of five.	Children's weight, height, BMI (crude and z scores), age in each measurement.	• Latent-class growth models<br>• Broken stick models<br>• SITAR models
3. Create and validate models to predict childhood obesity at the age of five.	*Predictors:* A set of predictors will be considered for the models – characteristics of the child, characteristics of the mother, and family/environment factors.<br><br>*Outcomes:* childhood obesity at the age of five and rapid infant growth at different ages (12, 18, 24, 36, 48, and 59 months).	Machine learning models using:<br>• Artificial Neural Networks<br>• Adaptive Fuzzy Systems
4. Estimate the effect of pre-pregnancy BMI, GWG, maternal weight, BMI changes between pregnancies, and maternal weight trajectories on adverse maternal and neonatal outcomes and child growth trajectories.	*Exposures:* Pre-pregnancy BMI, GWG, weight, and BMI changes.<br><br>*Outcomes:* LGA, SGA, LBW, macrosomia, preterm birth, mode of delivery, child BMI and growth indicators, child growth trajectories defined in goal 2, rapid infant growth, child mortality, and maternal mortality.	• Generalized linear models (Logistic and Poisson regression for categorical and linear and quantile regression for continuous outcomes)<br>• Linear mixed-effects models for longitudinal outcomes<br>• Confounding will be considered using directed acyclic graphs
5. Estimate the effect of pre-pregnancy BMI, GWG, and maternal weight and BMI changes on adverse maternal and neonatal outcomes in the subsequent pregnancy	*Exposures:* Pre-pregnancy BMI, weight, and BMI changes.<br><br>*Outcomes:* LGA, SGA, LBW, macrosomia, preterm birth, mode of delivery, children's BMI and growth indicators, growth trajectories defined in goal 2, rapid infant growth, neonatal and child mortality, and maternal mortality in the consecutive pregnancy.	• Generalized linear models (Logistic and Poisson regression for categorical and linear regression for continuous outcomes)<br>• Linear mixed-effects models for longitudinal outcomes<br>• Confounding will be considered using directed acyclic graphs
6. Estimate the effect of maternal and infant feeding practices on child nutritional status and growth trajectories.	*Exposures:* Food intake markers during pregnancy, breastfeeding, and complementary feeding.<br><br>*Outcomes:* child BMI and growth indicators, growth trajectories defined in goal 2, and rapid infant growth.	• Generalized linear models (Logistic and Poisson regression for categorical and linear regression for continuous outcomes)<br>• Linear mixed-effects models for longitudinal outcomes<br>• Confounding will be considered using directed acyclic graphs

Note: BMI: Body mass index; GWG: Gestational weight gain; SITAR: Super-Imposition by Translation and Rotation; WHO: World Health Organization.

For peer review only - http://bmjopen.bmj.com/site/about/guidelines.xhtml

For peer review only

FIGURE LEGENDS

Figure 1. Theoretical framework guiding the investigation of maternal and child nutrition within the 100 million Brazilian cohort.

For peer review only

For peer review only - http://bmjopen.bmj.com/site/about/guidelines.xhtml

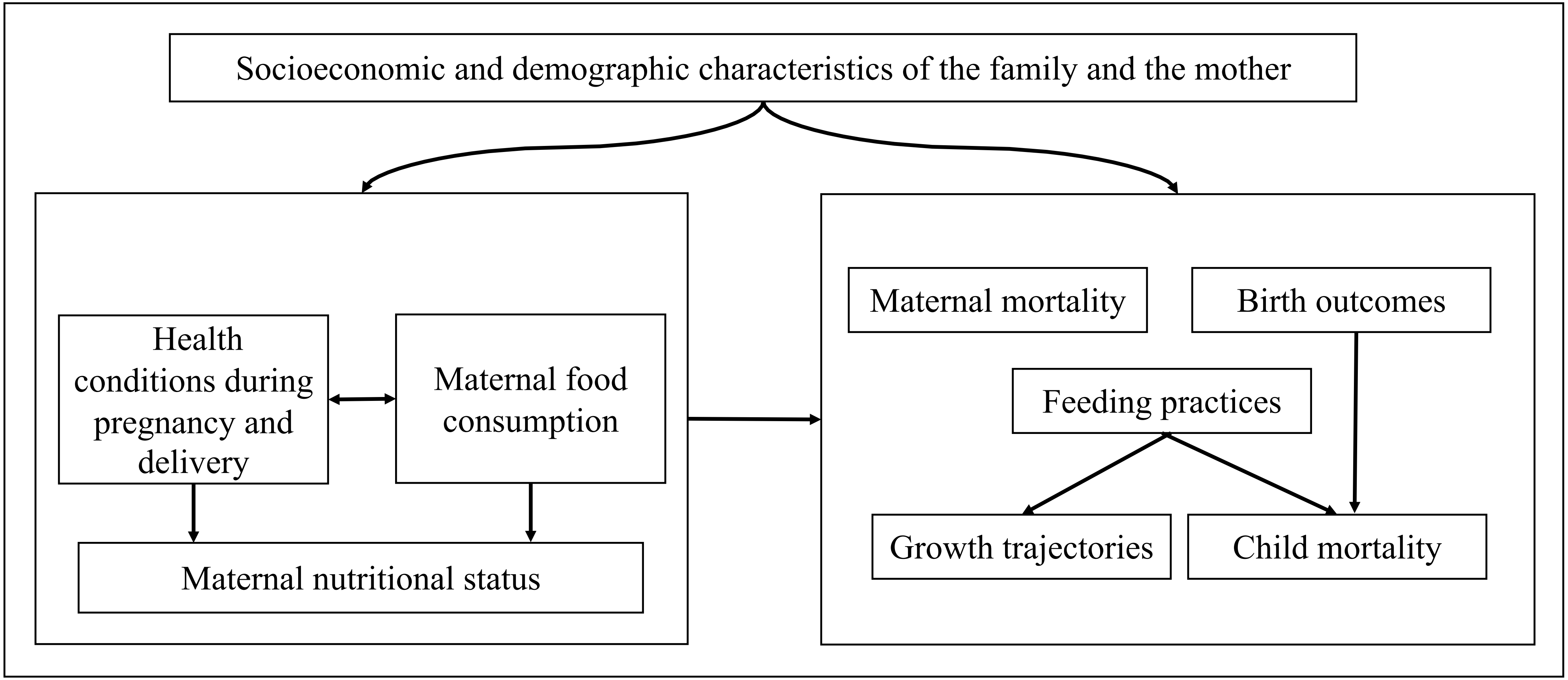
Socioeconomic and demographic characteristics of the family and the mother
Health conditions during pregnancy and delivery
Maternal food consumption
Maternal nutritional status
Maternal mortality
Birth outcomes
Feeding practices
Growth trajectories
Child mortality
